# A geo-chemo-mechanical study of a highly polluted marine system (Taranto, Italy) for the enhancement of the conceptual site model

**DOI:** 10.1038/s41598-021-82879-w

**Published:** 2021-02-17

**Authors:** F. Cotecchia, C. Vitone, F. Sollecito, M. Mali, D. Miccoli, R. Petti, D. Milella, G. Ruggieri, O. Bottiglieri, F. Santaloia, P. De Bellis, F. Cafaro, M. Notarnicola, F. Todaro, F. Adamo, A. Di Nisio, A. M. L. Lanzolla, M. Spadavecchia, M. Moretti, G. Agrosì, F. De Giosa, P. Fago, M. Lacalamita, S. Lisco, P. Manzari, E. Mesto, G. Romano, G. Scardino, E. Schingaro, A. Siniscalchi, G. Tempesta, E. Valenzano, G. Mastronuzzi, N. Cardellicchio, A. Di Leo, L. Spada, S. Giandomenico, M. Calò, V. F. Uricchio, G. Mascolo, G. Bagnuolo, R. Ciannarella, A. Tursi, G. Cipriano, P. Cotugno, L. Sion, R. Carlucci, G. Capasso, G. De Chiara, G. Pisciotta, R. Velardo, V. Corbelli

**Affiliations:** 1 DICATECh–Department of Civil, Environmental, Land, Building Engineering and Chemistry, Polytechnic University of Bari, via Orabona 4, 70125 Bari, Italy; 2 DEI–Department of Electrical and Computer Science Engineering, Polytechnic University of Bari, via Orabona 4, 70125 Bari, Italy; 3 CNR, IRPI, National Research Center, Via Amendola 122/I, 70126 Bari, Italy; 4 DISTEGEO, Department of Earth and Geoenvironmental Sciences, University of Bari Aldo Moro, Via Orabona 4, 70125 Bari, Italy; 5 CNR-IRSA, National Research Center, Water Research Institute, Via Roma 3, 74123 Taranto, Italy; 6 CNR-IRSA, National Research Center, Water Research Institute, Via F. De Blasio 5, 70132 Bari, Italy; 7 Department of Biology, University of Bari, Via Orabona, 4, 70125 Bari, Italy; 8 Special Commissioner for Urgent Measures of Reclamation, Environmental Improvements and Redevelopment of Taranto, Taranto, Italy

**Keywords:** Biogeochemistry, Ecology, Environmental sciences, Hydrology, Limnology, Solid Earth sciences

## Abstract

The paper presents the results of the analysis of the geo-chemo-mechanical data gathered through an innovative multidisciplinary investigation campaign in the Mar Piccolo basin, a heavily polluted marine bay aside the town of Taranto (Southern Italy). The basin is part of an area declared at high environmental risk by the Italian government. The cutting-edge approach to the environmental characterization of the site was promoted by the *Special Commissioner for urgent measures of reclamation, environmental improvements and redevelopment of Taranto* and involved experts from several research fields, who cooperated to gather a new insight into the origin, distribution, mobility and fate of the contaminants within the basin. The investigation campaign was designed to implement advanced research methodologies and testing strategies. Differently from traditional investigation campaigns, aimed solely at the assessment of the contamination state within sediments lying in the top layers, the new campaign provided an interpretation of the geo-chemo-mechanical properties and state of the sediments forming the deposit at the seafloor. The integrated, multidisciplinary and holistic approach, that considered geotechnical engineering, electrical and electronical engineering, geological, sedimentological, mineralogical, hydraulic engineering, hydrological, chemical, geochemical, biological fields, supported a comprehensive understanding of the influence of the contamination on the hydro-mechanical properties of the sediments, which need to be accounted for in the selection and design of the risk mitigation measures. The findings of the research represent the input ingredients of the conceptual model of the site, premise to model the evolutionary contamination scenarios within the basin, of guidance for the environmental risk management. The study testifies the importance of the cooperative approach among researchers of different fields to fulfil the interpretation of complex polluted eco-systems.

## Introduction

**Figure 1:**
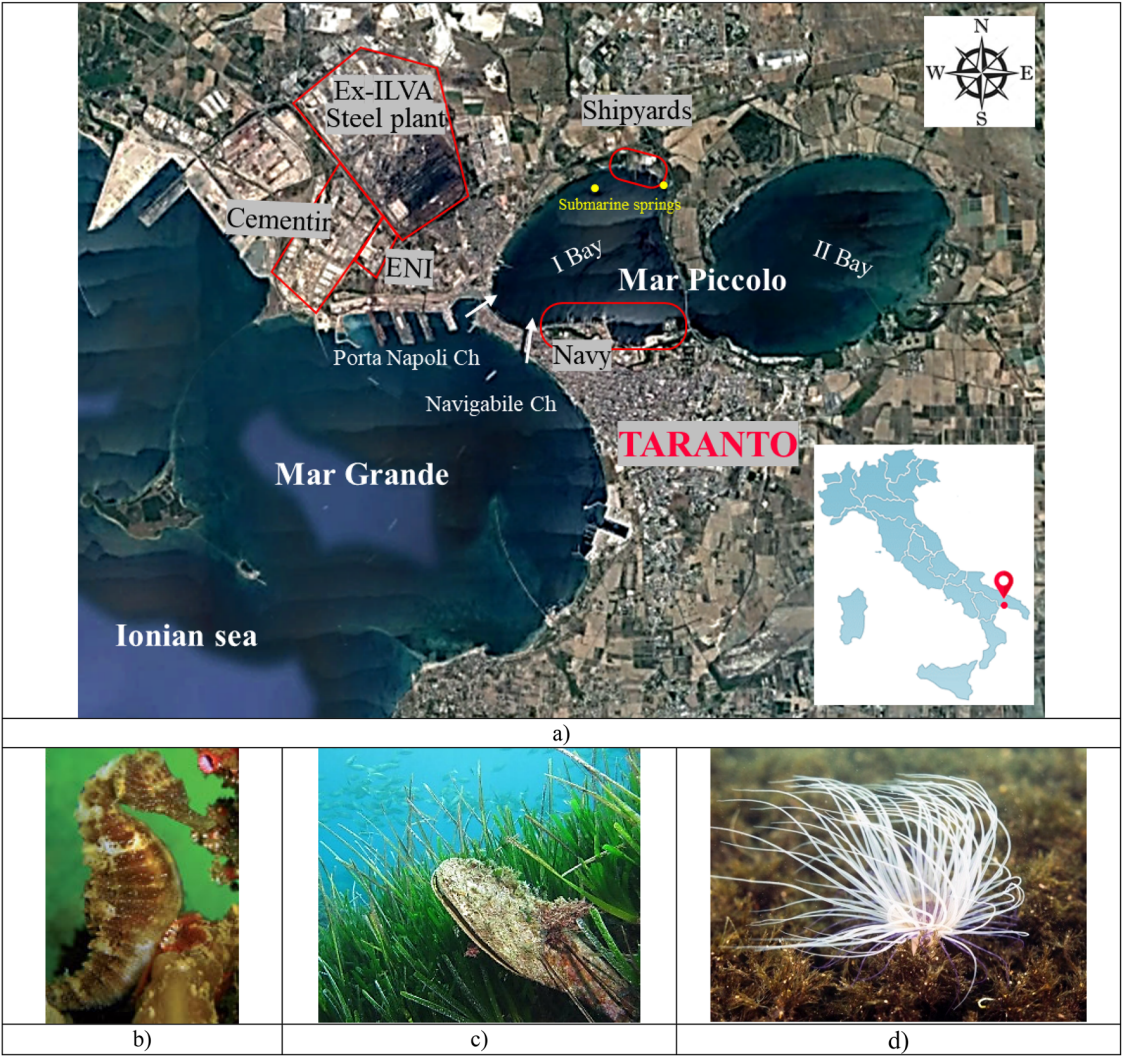
Gulf of Taranto in the South of Italy (**a**); (**b**–**d**) Photographs capturing the biodiversity in the Mar Piccolo and some typical protected species in the I Bay (e.g. seahorse, *Pinna nobilis, Sabella spallanzani* respectively). The map was obtained through Google Earth Software (https://www.google.it/intl/it/earth/) and further modified with Power Point Software—License to Polytechnic University of Bari (Italy). The photos of protected species (**b**), (**c**) and (**d**) are credits by Cipriano G. under CC BY open access copyright.

At present the town of Taranto (Southern Italy; Fig. 1) lies within such a highly polluted territory as to be included within the Italian Sites of National Interest, SIN^1^, which need urgently an environmental remediation. In particular, the Mar Piccolo (Fig. 1), in the northern part of the town, is a severely contaminated marine basin, of 20.72 km^2^ area, which includes two connected sheltered bays, the First (I) and a Second (II) Bay, of 12 m and 8 m maximum depth, respectively^2,3^. The I Bay is the only one connected to the open sea (Ionian Sea) through an open bay, the Mar Grande, via two channels, the natural “*Porta Napoli”* channel and the artificial “*Navigabile”* Channel (Fig. 1), excavated 130 years ago^4–7^.

The semi-enclosed features confer to the Mar Piccolo a limited sea water circulation. However, submarine springs (Fig. 1a), supplied by an underlying karst aquifer, recharge the Mar Piccolo with fresh water, which influences the equilibrium of its ecosystem and provides it with the features of a transition environment^8–10^. Such peculiarities have favoured, for several centuries, the farming of mussels and the living of protected marine species (Fig. 1b–d) in both the bays, till the onset of contamination in the last century.

The contamination has been logged in the sea water and in the marine sediments in the last two decades, in terms of metals and metalloids, e.g. As, Cd, Cr, Cu, Hg, Ni, Pb, and Zn, and persistent organic contaminants, e.g. polychlorinated biphenyls (PCBs), polycyclic aromatic hydrocarbons (PAHs), total hydrocarbons (volatile organic carbons, VOCs, and total petroleum hydrocarbons TPH), dioxins (polychlorinated dibenzo dioxins and dibenzo furans (PCDD/PCDF), excess of nutrients (N_tot_, P_tot_), which represent possible sources of high risk for the human health^11–18^. Such contamination has most probably developed progressively in the XIX century, as result of the uncontrolled discharge, in both bays, of the waste resulting from either industrial, or urban activities taking place in the surroundings.

As well-known, heavy metals, once introduced in the water column, may be adsorbed onto soil particles while these move towards the sea floor. The metals are then immobilized through either adsorption, or coagulation, or flocculation processes, sometimes even becoming part of the mineral structure of the sediments (e.g. Fe–Mn oxides^19,20^). Otherwise, the metals may precipitate generating insoluble fractions (such as metal sulphides). However, variations in the environmental conditions (e.g. pH, redox potential, microbial activity) may cause the leaching of the contaminants back to the water column, inducing their bioavailability, which poses a serious threat for the public health, given the toxicity of the contaminants, their persistent nature and possible biomagnification within the food chain^21^. The bio-availability of the contaminants may be also enhanced by either hydrodynamic dispersion, or remoulding and resuspension of the sediments, which add to leaching in representing the set of processes to be accounted for in the prediction of the contaminant fate and corresponding risk. In the case of the Mar Piccolo I Bay, the bio-availability of the contaminants has impacted on the various living species, including mussels^17,22^, to such an extent as to compel the banning of the mussel farming activities in 2011^23^.

In 2014, the *Special Commissioner for the urgent measures of reclamation, environmental improvement and redevelopment of Taranto* (Special Commissioner, hereafter), appointed by the Italian Government, promoted an advanced interdisciplinary study of the Mar Piccolo site conditions (i.e. water column and sediments, called system thereafter), to the aim of: (i) deepening the knowledge about the evolution with time of the site pollution; (ii) assessing the site environmental risk; (iii) identifying the Mar Piccolo portions requiring risk mitigation interventions; (iv) providing indications about possible sustainable remediation strategies. The present paper outlines the methodology adopted in this interdisciplinary study and discusses few of the results, in order to exemplify how its forefront aspects have prompted for the attainment of some of the aims listed above.

**Figure 2:**
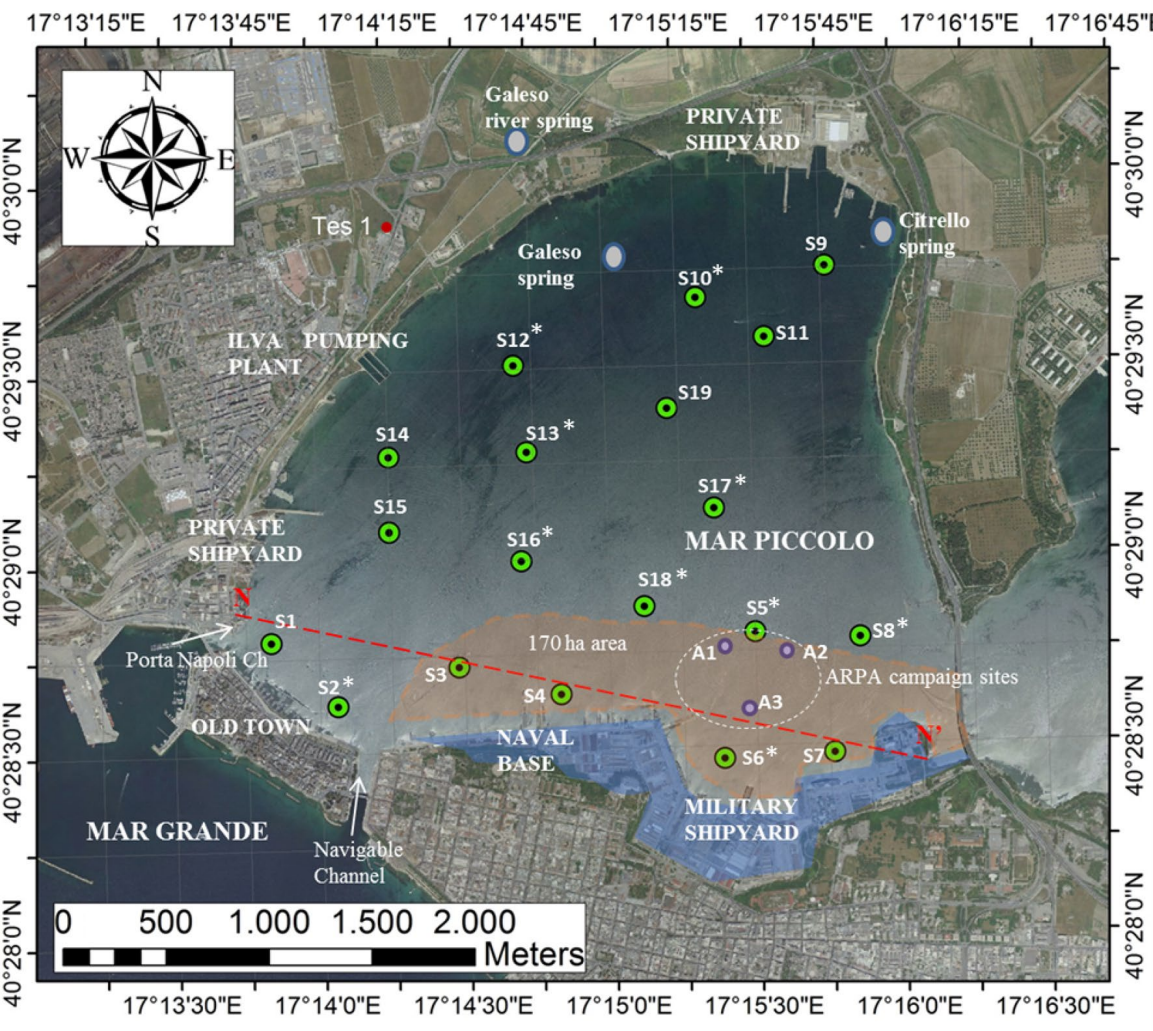
I Bay of the Mar Piccolo basin: boreholes and sampling sites of the 2017 investigation campaign promoted by the Special Commissioner (S sites) and of the campaign by ARPA in 2013 (A sites). Red dashed line indicates the trace of section N–N′. (*) sites equipped by piezocone tests. The map was obtained by co-authors through QGIS—software (version 3.14.16); https://eur03.safelinks.protection.outlook.com/?url=https%3A%2F%2Fwww.qgis.org%2Fen%2Fsite%2F&amp;data=04%7C01%7C%7Ca2eff60fe9454196008a08d8b0b20556%7C5b406aaba1f14f13a7aadd573da3d332%7C0%7C0%7C637453625938325668%7CUnknown%7CTWFpbGZsb3d8eyJWIjoiMC4wLjAwMDAiLCJQIjoiV2luMzIiLCJBTiI6Ik1haWwiLCJXVCI6Mn0%3D%7C1000&amp;sdata=J6w9DTllpaV5EynfaYk%2B2iGRAyDAimzXdOh6u10%2FXJQ%3D&amp;reserved=0), license Creative Commons. Attribution-Share Alike 3.0 licence (CC BY-SA) integrated with ESRI World Imagery.

Geologists, geophysicists, biologists, chemists, hydrogeologists, geochemists, mineralogists, geotechnical engineers and environmental technologists cooperated for 3 years in the research study, starting with the design of a cutting-edge investigation campaign in the Mar Piccolo I Bay (Fig. 2). They jointly designed sampling and testing strategies, sampling devices and sediment handling procedures, in order to ensure their compliance with the standards for the different testing fields. Thereafter, the whole team ended up with sharing an holistic and interdisciplinary interpretation of the contamination conditions, as advanced support to decision makers in the risk management of the specific site.

To start with, the system geological setting and the results of previous environmental investigations carried out in the I Bay^16,24^ are recalled in the following, since they represented the background of the new interdisciplinary study. Thereafter, some of the geological, chemical and hydro-mechanical data resulting from the new investigation are discussed, showing how their combined analysis allows to identify the factors which control the distribution of the contaminants and their mobility across the system. Subsequently, the conceptual site model is shown, as resulted from the analysis of all the results, and its role of technical strategic guidance in the selection of the risk remediation strategies is discussed.

Given the size of the database achieved through the whole study, the present paper discusses the results obtained from the testing of samples collected down to 45.5 m depth solely at six sites, S1, S2, S3, S4, S6, S7, which are aligned along the section N–N′ in the southern portion of the I Bay (Fig. 2). The data logged at the other sites will be presented in a following paper, which will outline the three-dimensional conceptual site model of the whole I Bay, according to the same methodology exemplified in the present paper for the two-dimensional conceptual site model of the southern portion of the I Bay.

## Overview of the geological and environmental features of the Mar Piccolo I Bay

### Geological and environmental setting

**Figure 3:**
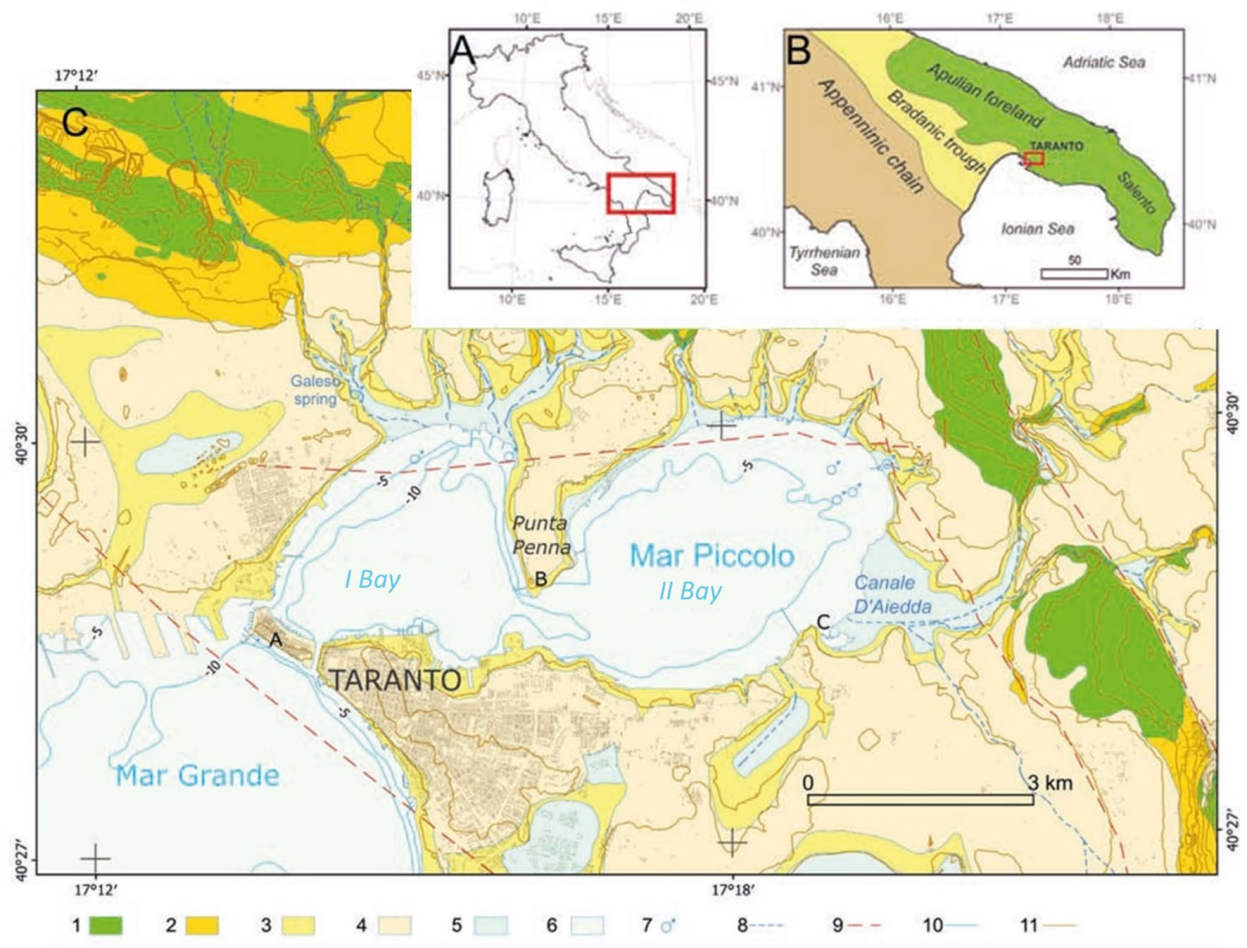
Geological map of the study area (C), whose location is shown in (A) and (B), modified after^29^. Key: 1—Altamura Limestone (*CA*, Cretaceous); 2—Gravina Calcarenite Formation (*GRA*, Upper Pliocene–Lower Pleistocene); 3—Sub-Apennine Clay Formation (*ASP*, Pleistocene); 4—Marine terraced deposits (*MTD*, MIS 5); 5—Alluvial deposits; 6—Holocene and Modern marine sediments; 7—Submarine springs; 8—Ephemeral drainage networks; 9—Buried faults; 10—Bathymetric contour, every 5 m; 11—Topographic contour, every 10 m (from^29^). The map in this figure was modified after Valenzano et al.^29^ with permissions of Comitato Glaciologico Italiano (https://eur03.safelinks.protection.outlook.com/?url=http%3A%2F%2Fgfdq.glaciologia.it%2F&amp;data=04%7C01%7C%7Ca2eff60fe9454196008a08d8b0b20556%7C5b406aaba1f14f13a7aadd573da3d332%7C0%7C0%7C637453625938325668%7CUnknown%7CTWFpbGZsb3d8eyJWIjoiMC4wLjAwMDAiLCJQIjoiV2luMzIiLCJBTiI6Ik1haWwiLCJXVCI6Mn0%3D%7C1000&amp;sdata=QwIiKWKItmzWIPKuZoeXd3mLAvZYrJLHi1lup8qRXDI%3D&amp;reserved=0 through QGIS—software (version 3.14.16); https://eur03.safelinks.protection.outlook.com/?url=https%3A%2F%2Fwww.qgis.org%2Fen%2Fsite%2F&amp;data=04%7C01%7C%7Ca2eff60fe9454196008a08d8b0b20556%7C5b406aaba1f14f13a7aadd573da3d332%7C0%7C0%7C637453625938325668%7CUnknown%7CTWFpbGZsb3d8eyJWIjoiMC4wLjAwMDAiLCJQIjoiV2luMzIiLCJBTiI6Ik1haWwiLCJXVCI6Mn0%3D%7C1000&amp;sdata=J6w9DTllpaV5EynfaYk%2B2iGRAyDAimzXdOh6u10%2FXJQ%3D&amp;reserved=0), license Creative Commons Attribution-ShareAlike 3.0 licence (CC BY-SA).

Taranto occurs about the eastern border of the Bradano Trough, just west of the Apulian Foreland (Fig. 3). In the study area, the Mesozoic carbonate basement of the Apulian Foreland underlies the Bradanic succession, formed of Plio-Pleistocene transgressive deposits. In turn, the succession is covered by Pleistocene to recent fine-grained sediments, of alluvial, to transitional, to marine origin^4,5,25–29^. The geological formations outcropping in the study are shown in the geological map reported in Fig. 3.

**Figure 4:**
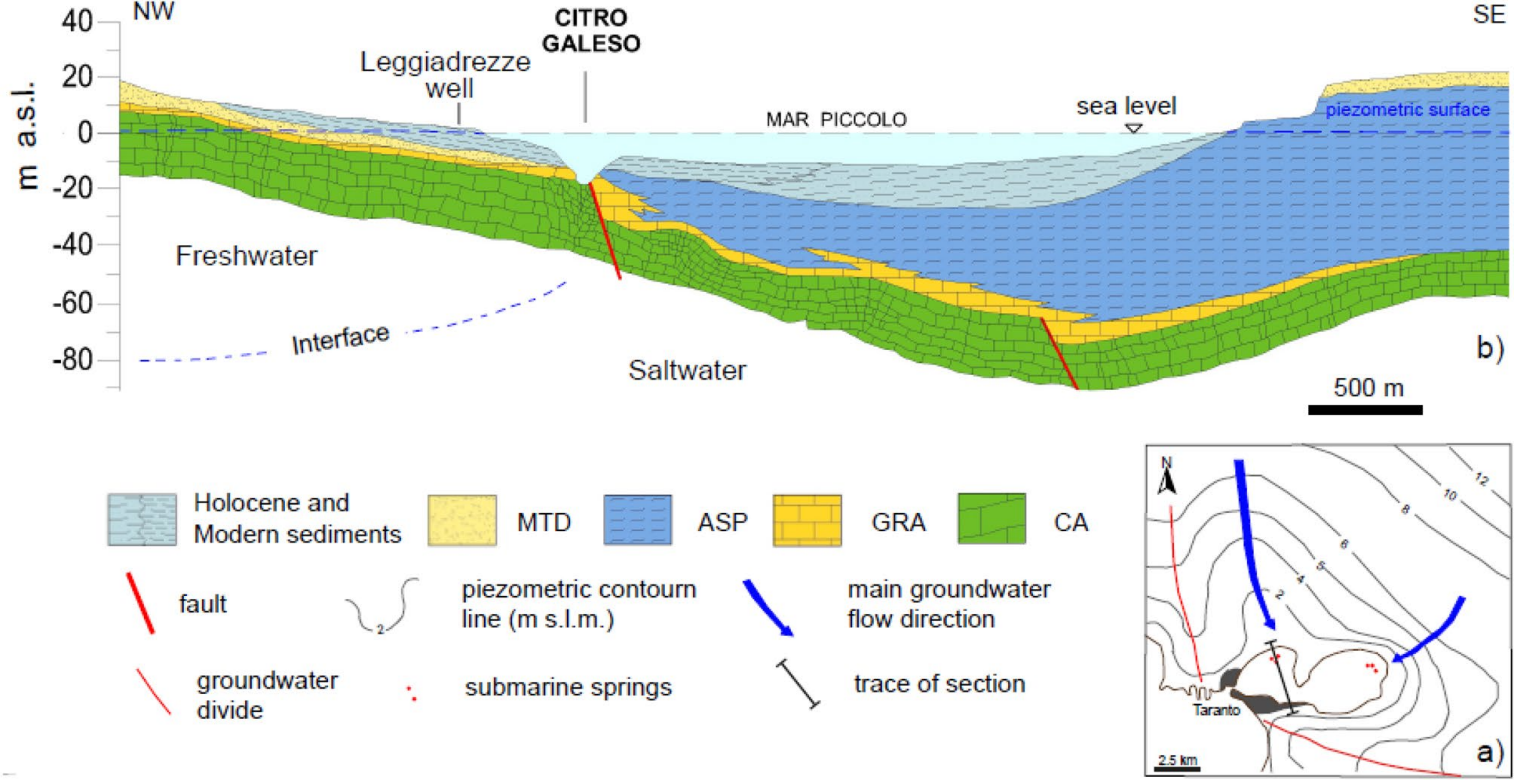
Map of the piezometric levels of the deep limestone aquifer (**a**) and hydrogeological sketch of section (**b**) whose trace is shown in the map b^25^. Key: CA, Altamura Limestone; GRA, Gravina Calcarenite Formation; ASP, Sub-Apennine Clay Formation, MTD, Marine terraced deposits. The map was obtained using Adobe Illustrator CNR VIP License—Annual Version.

Based on geophysical data^30^, reported that, at the Mar Piccolo sea floor, a thick stratum of soft Holocene sediments overlies the Pio-Pleistocene marine succession of the Bradano Trough (Fig. 4)^5,26–29,31–36^. Such Pio-Pleistocene marine succession is formed of the *Gravina Calcarenite* (*GRA,* Plio-Pleistocene), overlain by the *Sub-Apennine Clays (ASP,* Pleistocene*)*. *GRA* is a shallow water bioclastic calcarenite, of medium to high permeability. The *Sub-Apennine Clays* (*ASP*; Fig. 3c), which outcrop extensively in the Bradano Trough, are very stiff silty clays, rich in calcareous fossils. The upper part of this formation is highly weathered, due to physical–chemical processes^31,37^ and richer in sand levels. The *ASP* overly the *Altamura Limestone* (*CA*, Late Cretaceous), which is from calcareous to dolomitic and from medium to fine-grained, and it is permeable due to karst dissolution phenomena. Its top deepens from the northern (on average 20 m below sea floor, b.s.f.) to the southern part of the Mar Piccolo I Bay (reaching 80 m b.s.f.; Fig. 4). In both the Mar Piccolo Bays, alluvial and marine clayey-silty sediments deposited during the Late Pleistocene and the Holocene above the *ASP*. These are very soft at the sea floor^35^.

The morphology of both the Mar Piccolo bays resulted from Late Pleistocene to Holocene erosion processes alternating with sedimentation in marine-coastal, to river, to lagoonal and continental a deposition environments^4,34,38^. During the Last Glacial Maximum (MIS2 about 20 ka BP), the two bays were location of river erosion, before a new start of sedimentation occurred since the last Holocene marine transgression till present^4,5,29,39^. In recent times, also anthropogenic actions have recurrently caused local disturbance and remoulding of the very top soft sediments, affecting their sequence, especially in the southern part of the I Bay.

The *ASP* are characterized by a low coefficient of saturated permeability, i.e. k ≅ 10^–10^ m/s at the field scale^40,41^ and may underlie coarser soil deposits inland (e.g. Marine Terraced Deposit, MTD in Fig. 3), which may host phreatic shallow aquifers. At the same time, given their very low permeability, the *ASP* confine the groundwater flowing within the deep karst limestone aquifer, hosted in the *CA* platform. Such aquifer is recharged by the rainfalls infiltrating in the CA outcroppings inland and it is subjected to seawater intrusion in the coastal areas^42^. In the Taranto area, the piezometric heads in such deep karst aquifer drop from about 8 m above the sea floor (a.s.f.) at 10 km distance from the coastline, to about 1 m a.s.f. in the Mar Piccolo area (Fig. 4a)^30,43^ As shown in Fig. 4a, within such aquifer the freshwater flows towards the Mar Piccolo, where it is discharged through submarine springs (Figs. 1, 2, 3, 4) called ‘citri’, which occur at the seafloor where the *ASP* were eroded. This is, for example, the case of the submarine spring ‘citro Galeso’ shown in Fig. 4b^10,25^. According to the investigation commissioned by the Special Commissioner, the average flow over the year of 0.75 m^3^/s is measured at citro Galeso, whereas an average flow of 0.35 m^3^/s is found at citro Citrello (Figs. 2 and 4)^3,36,44,45^. Several other minor submarine springs occur in the I Bay, mostly in the north-eastern portion. In addition, various small tributary rivers discharge water in both the I and II Bays, through catchment areas location of either *CA*, *GRA* or *ASP* outcroppings. The Galeso river is the most important one discharging water in the I Bay, with a mean flow of 50,000 m^3^/day^3,36,45^, whereas the Canale D’Aiedda—Leverano D’Aquino provides the biggest river discharge in the II Bay (Fig. 3)^6,29,36^.

Since solely the I Bay is connected to the open sea through two channels (Figs. 1 and 2), on the whole the Mar Piccolo can be considered a sheltered sea of limited water circulation, where the tidal excursions do not exceed 0.30–0.40 m^36,46,47^. Furthermore, given the above cited discharges of freshwater (salinity about 3‰), the salinity of the I Bay seawater is about 35.07‰^48^. Hence, it is recognized that such hydrologic and hydrogeologic conditions make the Mar Piccolo environment representative of typical transition features^36^, with rich biocenosis and biodiversity. As represented in the map of Supplementary Figure S1), different species of algae, macroalgae (*Cladophora prolifera*, *Caulerpa prolifera*, *Chaetomorpha linum*, *Gracilaria dura* and *Dictyota dichotoma)* and species of high conservation value (Porifera *Geodia cydonium* and *Tethya citrina*, *Pinna nobilis, Hippocampus* and *H. guttulatus*)^49^ are present therein. Furthermore, both the I and II Bay are characterized by continuous fluctuations of nutrients, which determine high primary and secondary productivity. A first consequence of such a peculiar environment is the colonization of hard surfaces by suspensivorous and filter-feeding organisms, such as Ascidians, solitary and colonial, (*Phallusia mamillata*, *Clavelina lepadiformis*, *Distaplia bermudensis*), *Briozoans (Schizobranchiella sanguinea) and Polychetous (Branchiomma luctuosum* and *Sabella spallanzani*). Additional effect to be mentioned is the suitability of the marine site for the most important mussel farming plants in Europe, with an annual production of bivalves of about 40,000 tons per year^50^, lasted until 2011^23^.

### The onset of contamination

Since the second half of the XIX century, the town of Taranto and its coastline have become location of an intense industrialization. In particular, since 1889 the I Bay has hosted the largest naval base and military shipyard of the Italian Navy, along with other private merchant and fishery shipyards (Figs. 1 and 2). Furthermore, just west of the I Bay, a major oil refinery, ENI, and one of the largest cement and concrete plants in Southern Italy, CEMENTIR, have been operating since the second half of the XX century (Fig. 1). Lastly, since 1965 one of the biggest steel factories in Europe, named ILVA, has been active just 1.5 km north-west of the I Bay, pumping thousands of cubic metres of water off its north-western shore.

On the whole, the industrial, naval and urban activities have caused huge discharge of contaminants in the basin^13,15,51,52^, due to the scarcity of appropriate sewage treatments. Furthermore, chemicals deriving from the agricultural farming in the surroundings of the Mar Piccolo have been for long released in both the bays^3^. In particular, 14 uncontrolled sewage pipes discharging waste liquids in the basin have been identified during the recent investigations coordinated by the Special Commissioner.

**Figure 5:**
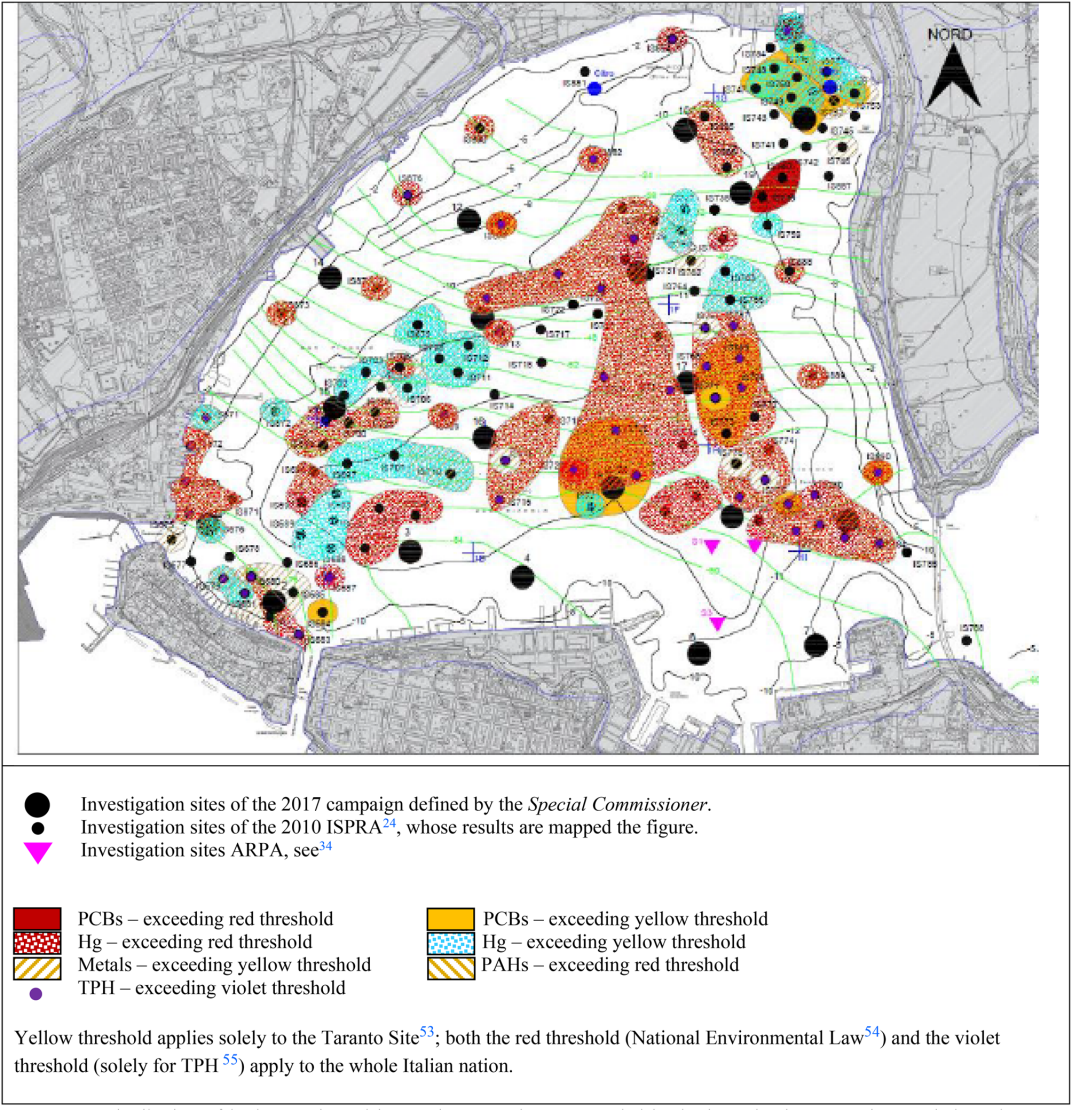
Distribution of both organic and inorganic contaminants recorded in the investigation campaign carried out by ISPRA (Institute for Environmental Protection and Research) in 2010^24^. The map was obtained through Autodesk AutoCAD 2017 Software (901-04337697 Licence).

Major chemical characterization campaigns were conducted in the basin between 2004 and 2013^16,24^. Their results revealed a high level of contamination for the Mar Piccolo sea water and sediments^51,52^. The map in Fig. 5 shows the distribution of both the organic and the inorganic contaminants recorded solely within the shallow sediments of the northern and central portions of the I Bay, by depths ranging from 0.5 to 3 m, in the campaign carried out in 2010 by the Italian Institute for Environmental Protection and Research (ISPRA), during which the southern portion of the bay was not explored. The contaminant concentrations have been mapped in the figure accounting for the corresponding threshold values set by the different regulations. In particular, the contaminant thresholds set by the regulations will be distinguished as follows: the threshold set for the Taranto Site of National Interest^53^ will be recalled as yellow; the threshold set for all the Italian industrial sites (National Environmental Legislative Decree^54^, not applying to TPH) as red; the threshold of Total Petroleum Hydrocarbons set within the National law^55^ as violet.

The mapping in Fig. 5 concerns the average contaminant concentrations logged in 2010 within the first 0.5 m depth at each of the investigation sites. Each coloured hatch is indicative of the degree of contamination for each contaminant, according to the figure legend. A uniform hatch covers multiple nearby sites when these were location of similar contamination degree. Conversely, when the contamination degree at a given site resulted different from that of the surrounding sites, a given hatch covers only a small area around the site. Such mapping procedure shows that high concentrations of one or more contaminants, either inorganic (Hg, As, Zn, Pb, Cr, Cu, Ni, Cd), or organic (PAHs, PCBs, TPH), exceeding the Taranto Site yellow threshold were present in large areas of the I Bay in 2010. In particular, Hg was the most widespread contaminant, even exceeding the National Environmental Law red threshold in the central portion of the I Bay. These high Hg concentrations were locally logged even down to 1.2 m depth at some sites. Moreover, there was a recurrent concentration above the yellow threshold for Pb, Cu and Zn. For the organic contaminants, concentrations exceeding the yellow threshold were logged for PAHs, in the South-West, Centre-West and North-East part of the I Bay, and for PCBs in the central and the Northern areas. Furthermore, PCBs concentrations exceeding the red threshold were logged in the North-Eastern part. The data testified also the widespread presence of high concentrations of TPH, even above the national violet threshold in an area close to the south-western coastline and in the central-eastern portion of the I Bay.

The 2010 investigation included also the measurement of the sediment organic content in terms of TOC. TOC values up to 8% were found within the shallow sediments in large part of the basin, as typically of coastal areas and estuarine muds (10%). However, such values are much higher than those typically measured in the shallow sediments of the Adriatic and Ionian Sea (i.e. less than 2%) and in the clay muds lying at the floors of deep seas (1–2%)^56^.

The hazardous contaminants were also found to impact the living species in the Mar Piccolo, since concentrations (Cd, Pb, PCBs and dioxins) exceeding the thresholds set by the European Community Regulation were recorded in fishes and mussels^17^.

A geotechnical characterisation of the soil profiles down to 18 m depth was carried out within a limited portion of the I Bay in 2013^16^ (Figs. 2 and 5), in front of the Military Shipyard. As reported by^35,57,58^, the geotechnical properties of the sediments occurring down to about 4–6 m depth were found to differ from those expected according to the soil skeleton composition. Such observation has suggested that the significant presence of organic matter and of either organic or inorganic contaminants in the sediments may be prompting coupled chemo-mechanical phenomena in the sediments, which modify their hydro-mechanical properties. Furthermore, the geotechnical investigation provided evidence of the presence of a few metre thick stratum of sediments of slurry consistency at the sea floor, within which remoulding processes could easily cause either the migration of contaminants from the sea floor down to few metre depth, or their resuspension in the water column.

Since the 2010 investigation (Fig. 5) had been strictly dedicated to the measurement of the degree and typology of contamination in the very top layers of the sediment deposit and did not provide indications about the potential migration over time of the contaminants towards the different environmental sectors of the system (i.e. the water column, the deeper sediment strata, the aquifers and the living species), the Special Commissioner issued the execution of the above cited new investigation campaign in 2017. Such campaign was planned to be multidisciplinary and to explore the whole sediment deposit (down to large depths) and its boundary conditions, in order to widen the knowledge about the distribution, mobility and availability of the contaminants across the soil deposit in the I Bay and, hence, reach the goals (i) to (iv) cited in the introduction section.

## The new multidisciplinary investigation

### The off-shore sampling campaign

The campaign was meant to investigate the sedimentological, geochemical, mineralogical and geotechnical properties of the I Bay seabed soil deposit, as well as the contaminant concentrations down to large depth. To start with, a geophysical survey of the basin was carried out before sampling, in order to achieve high resolution seismic profiles indicative of the variability of the sediments across the deposit and to steer the distribution of the sampling sites in I Bay. The geophysical survey allowed also to feature out the top of the *ASP* formation^29^, found to range between 5 m depth in the north of the I Bay (in land sampling site, named "Tes1", Fig. 2), up to more than 40 m depth in the southern part. The *ASP* were found to be locally absent in some northern portions of the I Bay, due to erosion.

Based on the geophysical survey results, 19 continuous coring sites were selected (Figs. 2 and 5). One of the main concerns was to take high quality sediment samples complying with the different testing standards holding in the different investigation fields. To this aim, the sampling techniques (Supplementary Fig. S2) and the sample storage were designed on purpose. Different samples were assigned to the different laboratories according to the outlined shown in Supplementary Fig. S3. For each of the sites, continuous coring was carried out down two twin boreholes, named A and B (Supplementary Fig. S3), 10 m far from each other. The sediments collected from each A borehole were tested in the environmental technology laboratory to investigate the effects of different remediation technologies for the contaminated sediments, after characterizing their contamination^59–61^. The sediments taken down each B borehole were used for the other tests. In particular, the continuous coring down the B boreholes was interrupted at given depths to retrieve undisturbed geotechnical samples^62^. In addition, at each site one undisturbed geotechnical sample was retrieved within the shallowest sediments by scuba divers (short borehole M in Supplementary Fig. S3), using on-purpose designed samplers (Supplementary Fig. S2).

The continuous coring machine was installed on an offshore platform (Supplementary Fig. S2), fixed in place through four poles of 40 cm diameter, stuck within the shallowest seabed sediments. Polycarbonate liners of 1.5 m long (chosen to avoid cross contamination phenomena) were used to sample the sediments subjected to sedimentological, geochemical, mineralogical and chemical tests. When brought to the laboratory, the liners were cut longitudinally in two parts; one portion (red colour in Supplementary Fig. S3a,b) was then used to retrieve the chemical samples, whereas the other portion (green colour in Supplementary Fig. S3a,b in SI) was used for the other tests. The undisturbed geotechnical samples were, instead, collected by means of samplers selected according to the sediment depth and consistency. Within the least consistent shallow sediments, of very high liquidity index^35,63–65^, thin-walled tube samplers (made of transparent polycarbonate), were pushed into the soil by the scuba diver (M boreholes in Supplementary Fig. S2a), whereas the sediments of medium consistency, at depths larger than 1.5 m, were sampled using the Osterberg hydraulic piston sampler. The length of the boreholes varied from 11.6 to 45.5 m; the bottom of the boreholes was always located at 1.5–3 m depth below the top of either the *ASP* or of the *GRA*.

### Sample storage, testing programmes and methods

Soon after sampling, the liners and the geotechnical samples were stored in a fridge on the off-shore platform, at + 4 °C, and then transferred to the laboratories. There, the liners were split as outlined above and stored at − 1 °C, for the geochemical, sedimentological and mineralogic tests, and at − 20 °C for the chemical tests.

Soon after extruding the sediment from either the liner or the geotechnical sampler, both pH and redox potential were measured. In addition, the organic matter content, OM, was determined for the sediments retrieved along the whole B boreholes, down to the *ASP* formation. For the chemical tests, the samples by 1.5 m depth were split in three sub-samples of 0.5 m height. The three sub-samples were mixed to form a representative homogenised and composite sample, which was subjected to the chemical testing. The chemical analyses were carried out also on small portions of the undisturbed geotechnical samples. The geotechnical samples were stored in the fridge in the Geotechnical laboratory.

The geological and sedimentological analyses were based on visual inspection of the sediment cores. The core description was performed focusing on the lithology, colour (Munsell soil colour chart), sedimentary structure, biological content (type and concentration of shells and organic material). High resolution photos were taken for each 20 cm long core, using a camera set in a fixed position, with an overlap of about 25% between subsequent photos. The results were recorded in stratigraphic logs, which were, thereafter, compared with the results of the geophysical investigations cited before, and with stratigraphic data from the literature^29,39^, in order to assess the geometry and the distribution of the sedimentary units across the deposit, and to interpret their geological origin.

The geotechnical testing programme included the characterization of: the soil granulometry, the geotechnical index properties, indicative of either the soil composition (Atterberg limits: liquid (w_L_) and plastic (w_P_) limits and soil specific gravity, G_s_), or the soil state (soil unit weight, γ, water content, w_0_, void ratio, e, degree of saturation, S_r_), the mechanical and the hydraulic parameters, such as the shear strength parameters, c′ and ϕ′, the deformability parameters, the undrained shear strength, S_u_, and the coefficient of saturated permeability, k. In particular, S_u_ was determined on the off-shore platform through pocket penetrometer tests on both the top and the bottom bases of the soil cores, when still in the liners, in order to assess the class of consistency of the cored sediments (Table 1)^66,67^. Furthermore, S_u_ was measured also through in-situ piezocone tests, performed at ten of the coring sites (reported with * in Fig. 2). All the tests were carried out according to the standard procedures set by American Standards Test Methods (ASTM) and British Standards (BS), adapted to take account of the presence of brackish fluid and contaminants in the soil, as well as of fragments of shells and fossils^58,68,69^. An integrated system (hardware and software), named GeoLab, for the geotechnical measurements was also developed^70^ in order to increase speed, accuracy, and productivity during testing. The developed software ensured the communication with different platforms used for data acquisition and allowed to follow remotely the testing results minimizing the exposition of the operators to the contaminated sediments.

**Table 1:** Soil consistency classification based on the undrained shear strength, S_u_ of an intact soil^66,67^.

Consistency	Description	S_u_, kPa
Very soft/fluid	Exudes between fingers when squeezed in hand	< 20
Soft	Moulded by light finger pressure	20–40
Firm	Can be moulded by strong finger pressure	40–75
Stiff	Cannot be moulded by fingers Can be indented by thumb nail	75–150
Very stiff	Can be indented by thumb nail and crushable under pressure	150–300
Hard	Cannot be indented by thumb nail	> 300

The geotechnical characterisation of the sediments was aimed at the definition of the geotechnical model of the basin (first conceptual, quantitative thereafter), of use for the prediction of: the sediment susceptibility to either remoulding or resuspension; the hydrodynamic dispersion of the contaminants through the sediments (by means of numerical modelling); the sediment settlements for different possible remediation measures (by means of numerical modelling). In the following, though, the discussion will concern solely those soil features and parameters useful to: define the conceptual geotechnical model of the system; foresee the attitude to remoulding and resuspension of the sediments; foresee the attitude of the contaminants to undergo advective mobility through the deposit; estimate the qualitative features of the sediment responses to alternative remediation strategies, such as dredging and capping. To these aims, the discussion will deal solely with the sediment granulometries, index properties, coefficients of permeability and undrained shear strengths. The discussion of all the other geotechnical parameters, together with both the numerical modelling of the hydrodynamic dispersion of the contaminants through the sediments and the modelling of the geotechnical response of the system to different remediation interventions, will be covered in a subsequent paper, given the space that the presentation of these modelling applications and results requires.

The chemical tests were addressed to the determination of the concentrations in the sediments of those contaminants found to be of high concern in the previous investigations^13,15,24,51,52^. In particular, the concentrations of the metals and metalloids: Al, As, Cd, Cr, Cu, Fe, Hg, Mn, Ni, Sb, Ni, Pb, and V, of the persistent organic pollutants: PAHs, PCBs, PCDD/PCDFs, VOCs, TPH, polybrominated diphenyl ethers (PBDEs), organotin (OTs) and organo-chlorinated pesticides, and of the organic matter (OM), were measured, according to the appropriate standardized procedures. EPA methods 3052 (ICP/MS)^71^ and 8270D (GC/MS)^72^ were used for metals and PAHs, respectively. The concentrations of PCBs, PCDD/PCDFs and PBDEs were measured according to the methods CEN 15308^73^, EPA1613B^73^ and UNIEN16377^74^ respectively, and the tests were performed through gas chromatography/tandem mass spectrometry (GC/MS–MS). VOCs were determined according to EPA8015D method^75^, while for TPH (C_12_-C_40_) the method was ISPRA 75/2011 (GC-FID)^76^. Pesticides were measured according to Environmental Protection Agency (EPA) Method 8270D (GC/MS/MS)^72^, and OTs were determined through ICRAM 2001^77^. Finally, the OM contents were characterized through the Loss On Ignition method (LOI, i.e. combustion in an unheated muffle furnace, according to EPA 160.4^78^). In the following, the discussion about the chemical data will focus only on the concentration of nine trace elements (As, Cd, Cr, Cu, Hg, Ni, Pb, V and Zn) and of PAHs, (in terms of sum of 16 EPA PAHs congeners), PCBs (in terms of sum of 31 PCB congeners), TPH and OM contents (Supplementary Table S1).

The geochemical analyses were carried out through tests performed soon after opening the liners. The concentrations of As, Cu, Cr, Ni, Pb and Zn were measured using an on-purpose designed Portable X-Ray Fluorescence (PXRF), calibrated according to standards NIST^79–82^; furthermore, the magnetic susceptibility was also measured. The results have allowed to correlate the metal concentrations and the magnetic susceptibility with the litho-stratigraphic features observed through both the geological and the sedimentological analyses. The geochemical data obtained by PXRF were compared with the metal concentrations obtained using the EPA methods^71^, with the aim at finding evidence of how, for each given metal, the chemical concentration measured as average over a depth interval (EPA methods) derives from the distribution of local metal concentrations across the sediment.

Mineralogical analyses were carried out by means of X-Ray Powder Diffraction (XRPD) on several geological samples, as well as on the specific sediment intervals where geochemical anomalies had been detected. Handling and treatment of sediment samples for the mineralogical analyses were performed according to the United States Geological Survey (USGS) protocol. The data acquisition and interpretation complied with what reported by Hiller^83,84^. The composition of the clay fraction was analysed in the form of random mount, as well as of oriented mounts, subjected to various treatments (air drying, glycolation, heating at 400 °C and at 550 °C).

**Figure 6:**
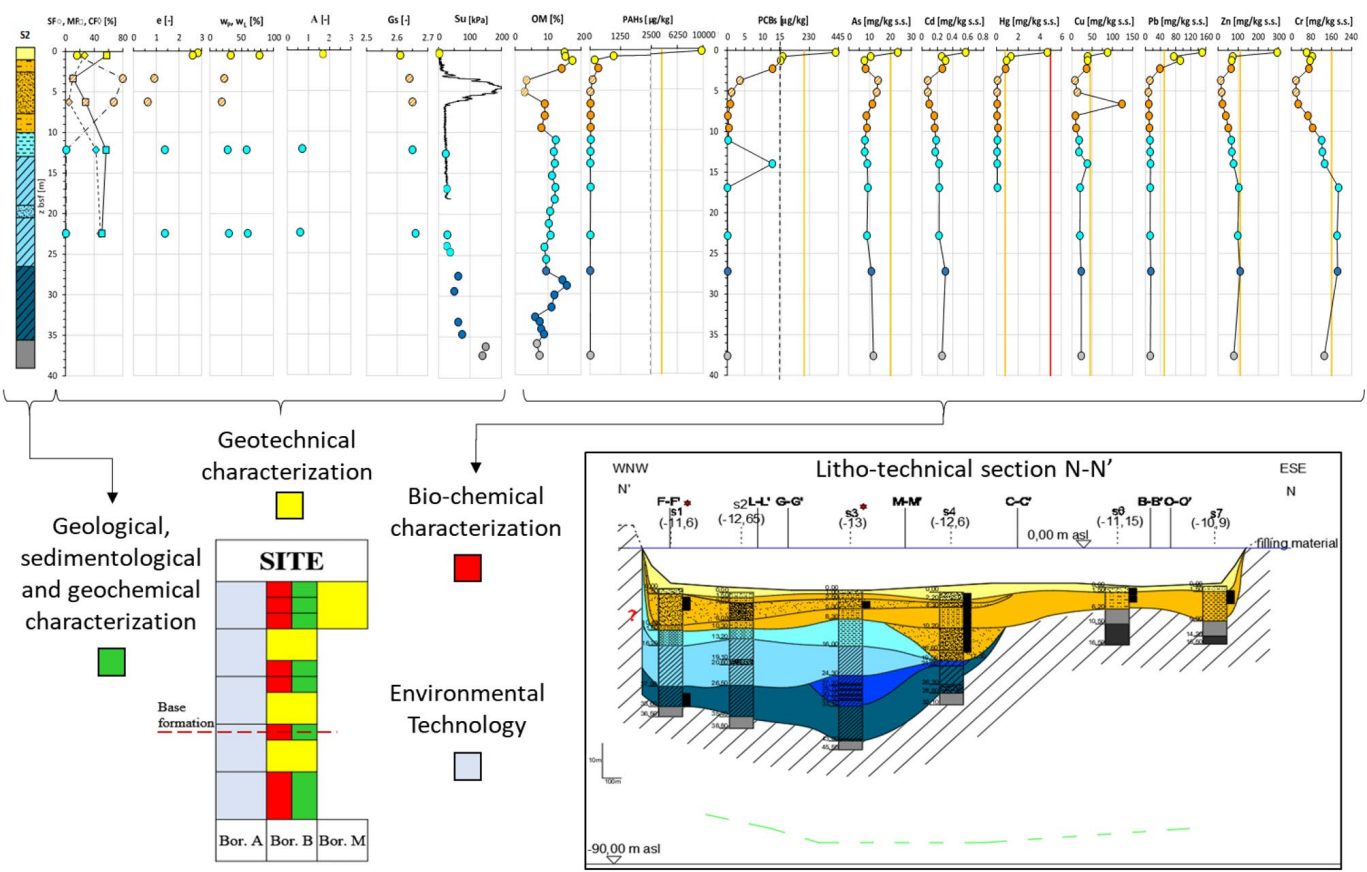
Schematic outline of the combination of the results from the different testing fields performed in the sediments retrieved in the investigation campaign.

Figure 6 exemplifies the combination of the results of all the different tests cited above.

## Discussion of the results

### The Litho-technical characterization of the deposit

**Figure 7:**
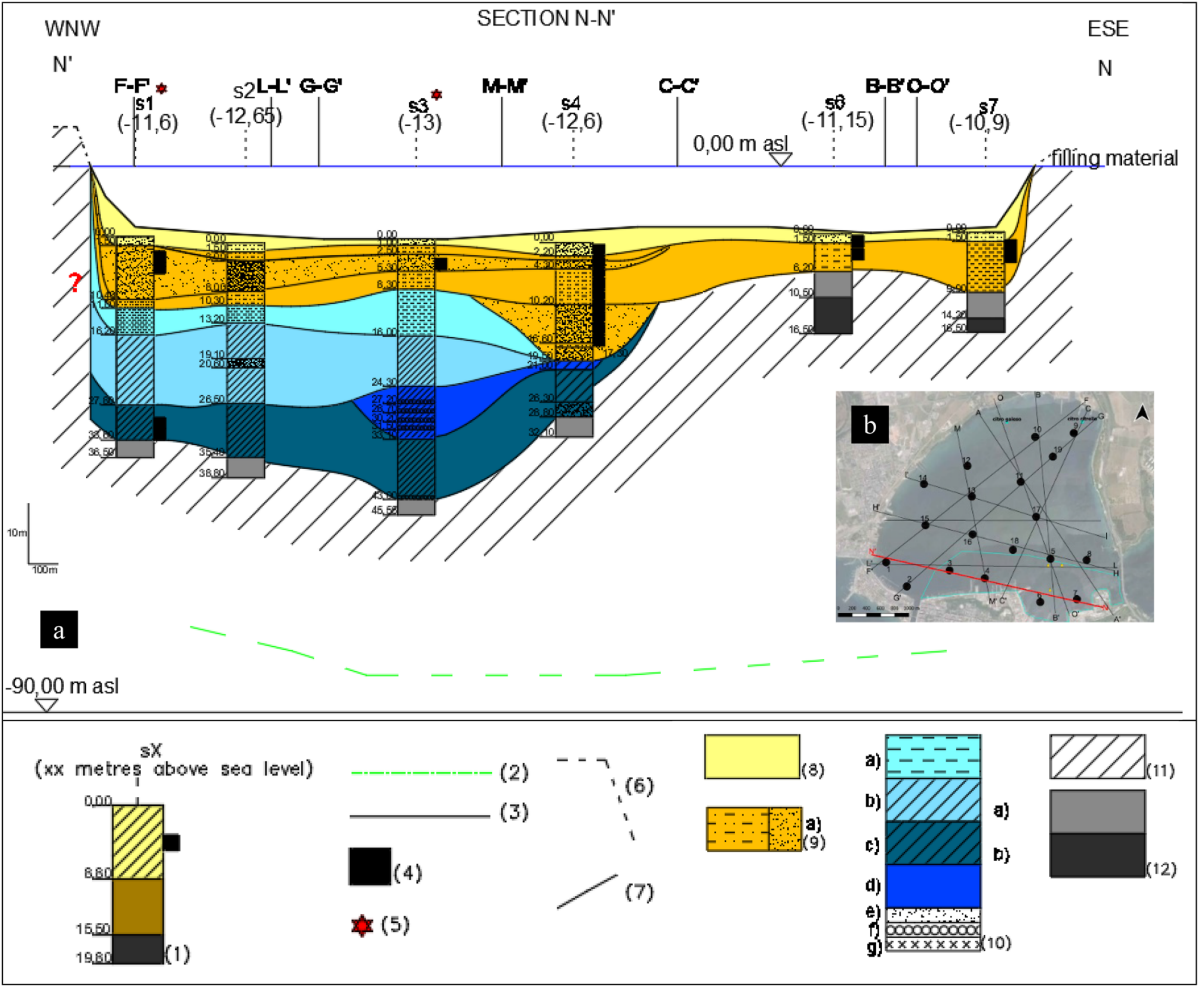
(**a**) Litho-technical section N–N′; (**b**) I Bay and location of all the investigated sections. Key: (1) 2017 campaign projected borehole; (2) top of the calcareous bedrock according to^30^ (3) bathymetry (Port authority 1947–1978); (4) significant content of organic matter; (5) fishing net (anthropogenic material); (6) coastline; (7) stratigraphic contact; (8) *1stLTU*; (9) *2ndLTU*, of consistency from very soft to soft and occasional presence of sand or silty sand, from very loose to loose (**a**); (10) *3*^*rd*^*LTU*, of consistency increasing with depth, from very soft to soft (**a**), from soft to firm (**b**), firm (**c**), stiff (**d**)^66,67^, and occasional layers rich in sand (**e**), gravel (**f**) and peaty levels (**g**); (11) Possible disturbed top layers of the *ASP* formation; (12) *ASP* formation, with clayey silt or silty clay of very stiff consistency, and sandy levels (S_u_ = 200–500 kPa) (**a**), or Grey-bluish marly-silty clay (S_u_ > 500 kPa) (**b**).

The litho-technical characterisation of the sediments has resulted from: the geological inspection of the cores in the liners and of the undisturbed geotechnical samples; the paleogeographic reconstruction of the soil deposition^29,39^; the soil geotechnical index properties; the geochemical and the mineralogical analyses. Here-forth, Fig. 7a reports the litho-technical section N–N′ whose trace is shown in Fig. 7b.

**Figure 8:**
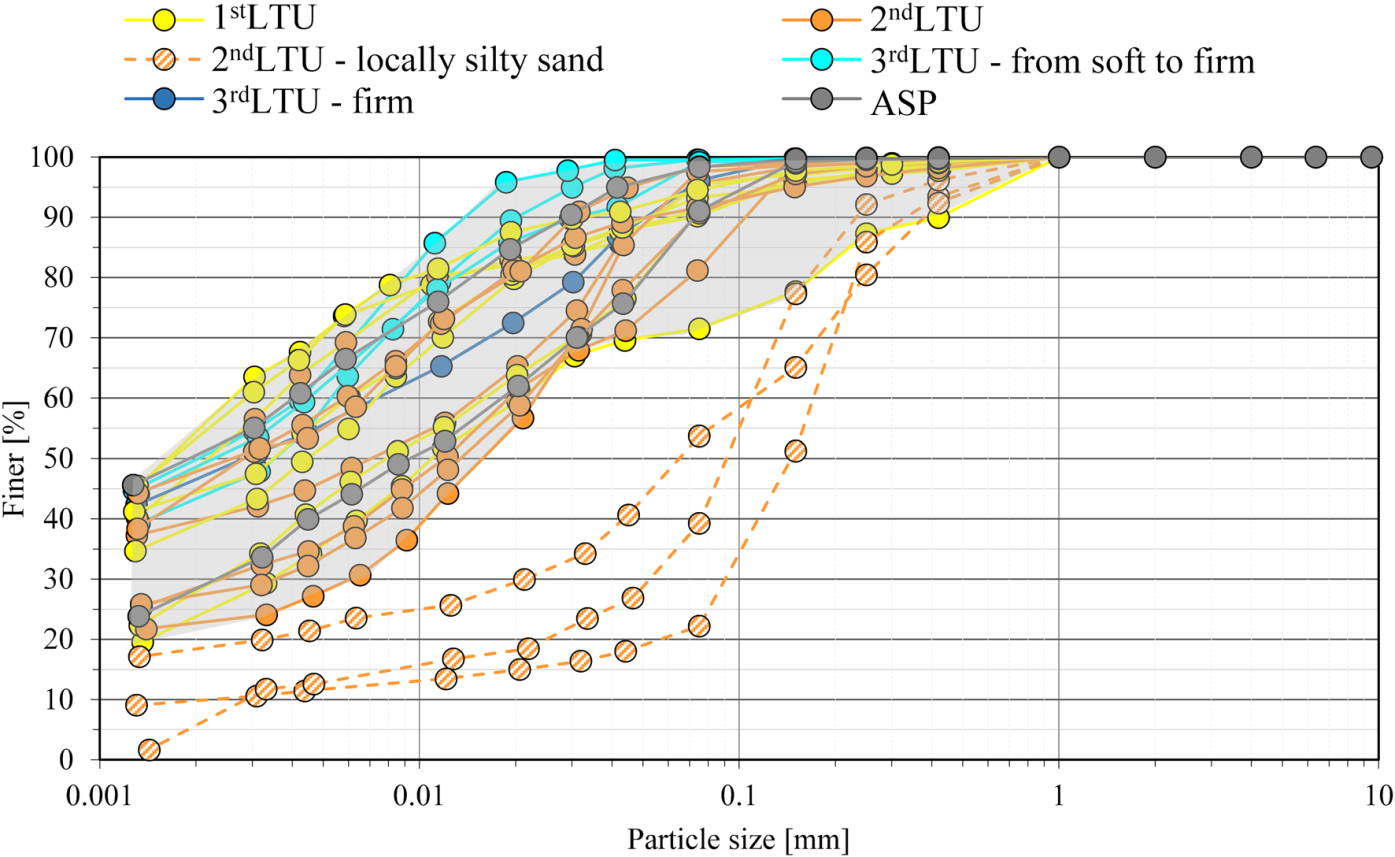
Grading curves of the Mar Piccolo sediment samples collected within different LTUs (S1, S2, S3, S4, S6, S7 sites of section N–N′ in Fig. 2).

A *First litho-technical unit*, hereafter *1*^*st*^*LTU* (light yellow colour in Fig. 7a), of about 1.5 m thickness, has been found to cover the whole deposit. It is formed of either clay with silt, or sandy to slightly sandy silt with clay, deposited in recent times up to present, according to the sedimentology and paleogeographic studies. The corresponding grading curves (Fig. 8) show that its clay fraction, CF, varies in the range 27–53%, its silt fraction, MF, in the range 39–57%, and its sand fraction, SF, is minor, except for site S1, close to the Porta Napoli channel (Fig. 2). It is rich in organic matter and the pocket penetrometer S_u_ data (S_u_ < 20 kPa) prove its largely fluid consistency (Table 1). The coefficient of permeability, K, measured by oedometer testing on the samples taken by scuba divers within this unit (M boreholes), varies in the range 10^–8^–5 × 10^–9^ m/s.

Given the very low consistency of the *1*^*st*^*LTU* sediments, it is very likely that these have been either remoulded, or resuspended all way through their history, due to the navigation activities in the south of the I Bay and to the dragging of ship anchors (Figs. 1 and 2). Furthermore, Mastronuzzi et al*.*^7^ provided evidence of the occurrence of important flooding events in the last two centuries (1883, 1996 and 2005), causing resuspension and redeposition of the 1st *LTU* sediments. Given so, the exact age of the sediments within this unit cannot be assessed.

The underlying *Second litho-technical unit*, hereafter 2nd *LTU* (light orange colour in Fig. 7a), is on average 6 m thick and it is formed of grey-coloured sandy or clayey-sandy silt, or clay with silt, of consistency varying from fluid to soft (S_u_ =  < 40 kPa) and permeability, K, on average about 10^–9^ m/s, if a sand layer occurring in the western part of the N–N′ section is excluded (Fig. 7a). Accordingly, the grading curves of this unit show that SF ranges between 3 and 24%, whereas CF and MF vary in the intervals 22–39% and 43–59%, respectively (Fig. 8), except for the sand layer interbedded in this unit in the western part of section N–N′ (SF = 52.8–79.8%; Fig. 8). For this sand level, from medium-dense to loose, an average K = 10^–6^ m/s has been measured by means of permeameter testing^85^. According to both the paleo-geographic and the sedimentological analyses, also the 2nd *LTU* sediments, slightly coarser than the *1*^*st*^*LTU* sediments, are either present day or recent and derive from the erosion of calcarenites and parent coastal-alluvial, or marine formations in-land.

At larger depths, several boreholes cross a *Third litho-technical unit,* hereafter 3rd* LTU* (light to dark blue colours in Fig. 7a). This is formed of silt, clayey silt or silty clays (Fig. 8), including local sandier levels, gravel levels and peat. It is of lower permeability than the overlaying units (K = 10^–9^–5 × 10^–10^ m/s) and includes different sub-units. The lower portion of the 3rd *LTU* is interpreted to be the result of a high energy fluvial deposition, most probably occurred during the Last Glacial Maximum (33–14 ka)^86^. Above the fluvial sediments, the recognition of levels of peat with pulmonated gastropods suggests that part of this unit was deposited within a transient continental environment, most likely at the beginning of the Holocene (11–10 ka). This part of the 3rd *LTU* passes gradually to upper silts and clays containing lagoonal shells, which mark the beginning of the marine transgression, within a sheltered marine environment. About 9 ka years ago, this sheltered marine basin was affected by the fall of volcanic ashes, deriving from the Pomici di Mercato eruption of Vesuvius. As a result of such event, a whitish porous tephra layer, 5 to 40 cm thick, is found to occur locally within this unit, at about 19–21 m depth, as confirmed by the results of PXRF analyses discussed later.

The consistency of the 3rd *LTU* is minimum for the first very soft sub-unit (light blue in Fig. 7a; S_u_ < 20 kPa; Table 1), and higher at medium depth, in the soft second sub-unit (blue in Fig. 7a; S_u_ = 20–40 kPa; Table 1). Underneath, the sub-unit shown as dark blue in Fig. 7a is of firm consistency, i.e. S_u_ = 40–75 kPa, and overlays the deepest stiffest sub-unit, i.e. S_u_ = 75–150 kPa (Table 1), shown in cobalt blue. Boreholes S6 and S7 (Fig. 7a) do not cross the 3rd *LTU*, since the 2nd *LTU* overlies directly the *ASP.*

The *ASP* have been found at the bottom of all the boreholes in the section (Fig. 7a). They are grey-bluish silty clays (Fig. 8), from very stiff (S_u_ = 150–300 kPa, light grey, Table 1) to hard (S_u_ > 300 kPa, dark grey, Table 1), of low permeability (k = 10^–11^ m/s laboratory measurement, k = 10^–10^ m/s field measurements). Their top is deepest in the southern part of the I Bay and deepens to the west of the section (Fig. 7a)^29^. The irregular surface of the *ASP* top represents the result of river erosion during the Last Glacial Maximum, before the deposition of the overlying units^29,39,87^. Neither the *GRA,* or the *CA* (Figs. 3 and 4) were reached in the drilling operations along section N–N′; the top of *CA* reported in Fig. 7a (dashed green line) is that inferred by^30^ through geophysical surveys.

**Figure 9:**
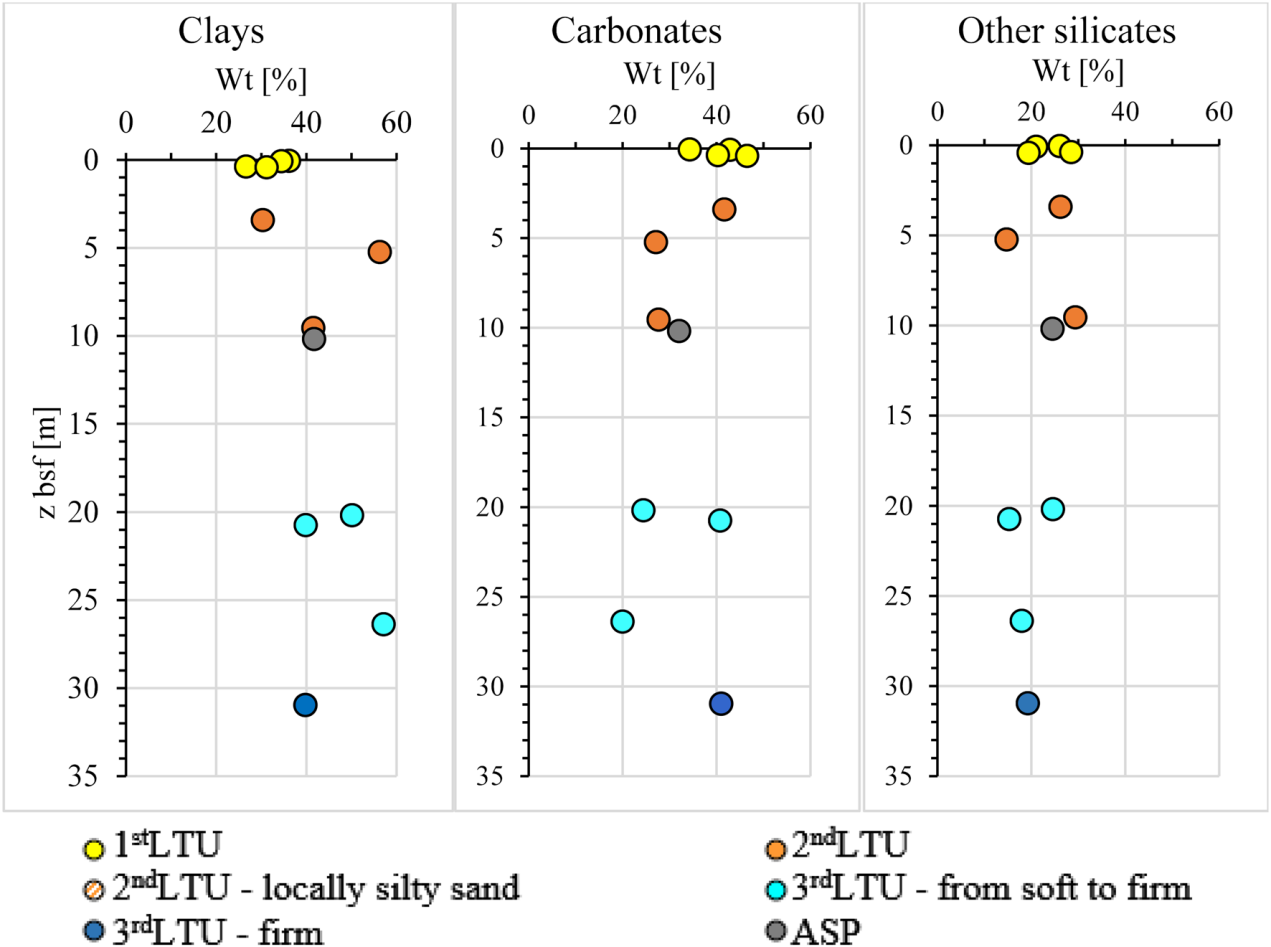
Profiles of mineralogical composition of the samples collected within different LTUs (sites S1, S2, S3, S4, S6, S7 of section N–N′ in Fig. 2).

The XRPD analyses, carried out on the S1 to S7 corings have shown that all the sediments have similar mineralogical composition to the *ASP* clays^41,88–92^. As an example, Supplementary Fig. S4 illustrates the diffraction XRPD patterns for samples from borehole S2. The main mineralogical phases detected in the analyses are: clay minerals, quartz, carbonates (mainly calcite and aragonite), plagioclase and feldspar. Minor phases also occur, but they are not ubiquitous and are not homogeneously distributed within the sediments. The vertical profiles of mineral content in Fig. 9 testify an increase of clay mineral content with depth. This ranges from 24 to 40% in the 1st *LTU*, and from 40 to 60% in the 3rd *LTU.* In the 2nd *LTU*, the clay mineral content reaches an out of trend peak of 56% in a sample at 5 m depth b.s.f. in borehole S1, where an amorphous component is abundant and impacts the accuracy of the determination. The clay minerals are a mixture of illite, chlorite, kaolinite and inter-stratified illite–smectite phases (I-S); the smectite minerals are not detectable in the diffraction patterns as a single mineralogical phase.

The pore water salinity has been found to range between 30 g/L and 36 g/L within the *1*^*st*^*LTU*, and to be about 32 g/L within the deep sediments of the 3rd *LTU*^58^. Therefore, the salinity of the pore water in th Holocene sediments remains high with increasing depth.

**Figure 10:**
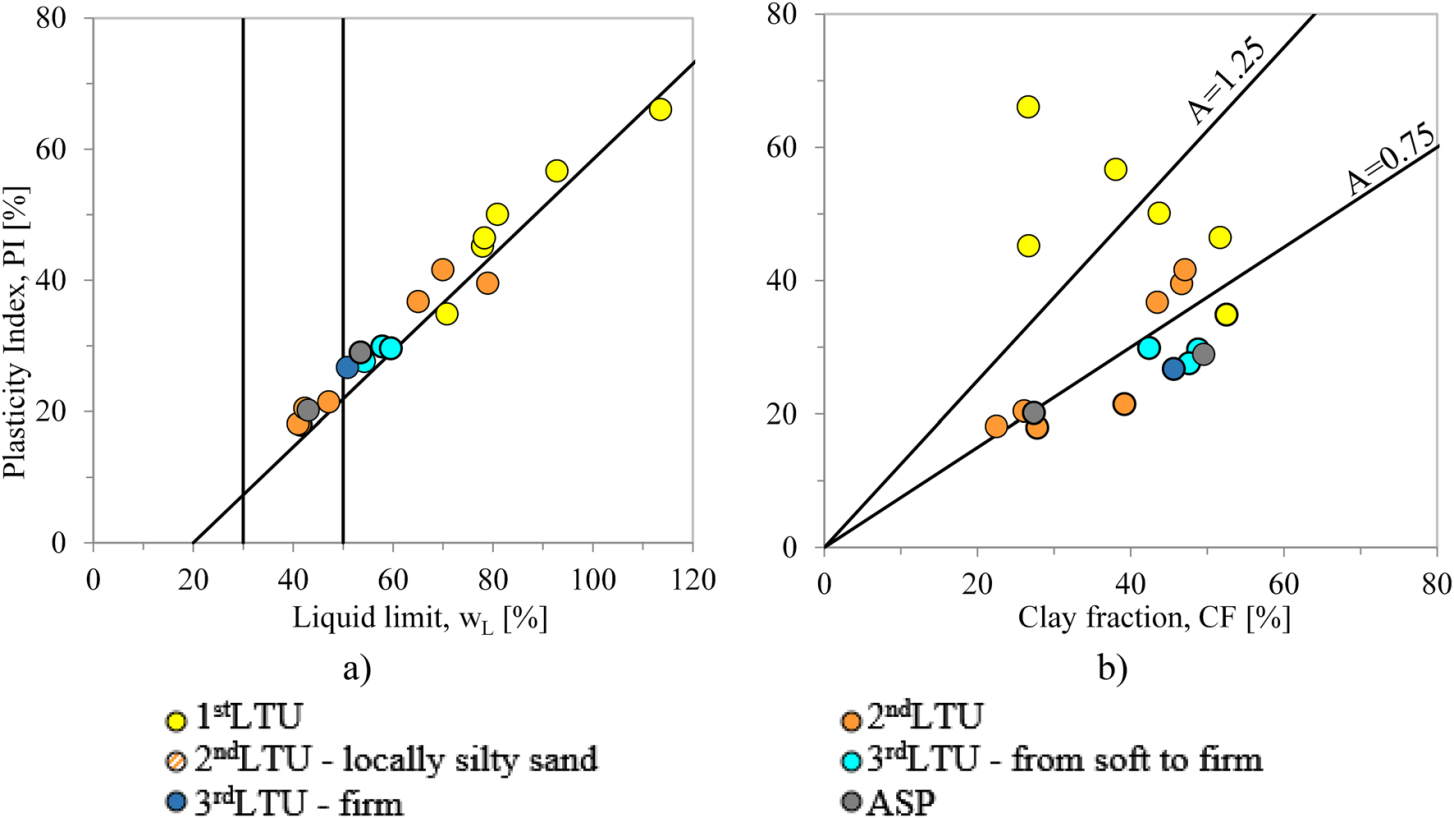
Casagrande’s plasticity (**a**) and Activity (**b**) charts of the samples collected within different *LTUs* (S1, S2, S3, S4, S6, S7 sites of section N–N′ in Fig. 2).

The limited variability in both the granulometry and the mineralogy of the sediments forming the different units, except for the coarser sediments locally interbedded in confined levels, is not consistent with the significant differences among the values of some geotechnical index properties recorded for the different units. This is the case for the values of the liquid limit, w_L_^93^, the plasticity index, PI (PI = $\left( {{\text{w}}0 - {\text{wP}}} \right)$)^93^ and the activity index, A = PI/CF, measured for the 1st *LTU* and 2nd *LTU* on one side, and those characterizing both the 3rd *LTU* and the *ASP*, on the other. Such differences are evident in Fig. 10, reporting the data in the Casagrande plasticity chart (Fig. 10a) and the activity chart (where PI is plotted versus the clay fraction CF, to characterize the activity index A; Fig. 10b). It is worth remarking that since w_L_, PI and A are the geotechnical indices most closely related to the amount and mineralogy of the clay fraction, CF, and to the pore water salinity, their values should vary little among the 1st to the 3rd* LTU*s and the *ASP*. Conversely, the samples belonging to the 1st *LTU* are characterised by values of w_L_ (70–113%), PI (35–66%) and A (> 1.1) much higher than those recorded for the 3rd *LTU* and the *ASP.* Such anomaly is the first evidence of the dependence of the geotechnical properties of the sediments present in the I Bay on their content in contaminants and OM, as confirmed in the following.

### Chemo-mechanical features

**Figure 11:**
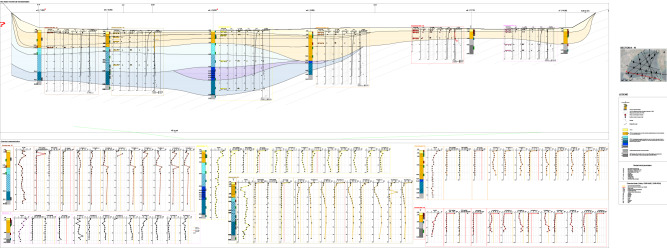
Geo-chemo-mechanical section N–N′_GCM_.

The integration of all the data acquired along section N–N′ (“The new multidisciplinary investigation” section; Fig. 6) is exemplified in Fig. 11. As such, the figure represents the geo-chemo-mechanical section N–N^′^_GCM_ of use for the analysis of the spatial variability across the deposit of the lithological and geotechnical soil properties together with the contaminant concentrations. For each site the figure reports the litho-technical profile, aside the profiles of the soil granulometry and geotechnical properties: e_0_, w_L_ and w_P,_ A, Gs, S_u_. These profiles can be compared with those of the concentrations in: As, Cd, Cr, Cu, Hg, Ni, Pb, Zn, V, PCBs (expressed in term of sum of 31 PCB congeners), PAHs (expressed in terms of sum of 16 EPA congeners), TPH, and the OM content profile. All the data are plotted at the average depth of the sample they correspond to. Furthermore, for each contaminant profile both the threshold value set for the Taranto Site (yellow line) and that set by the National Environmental Law for industrial sites (red line) are reported. Such threshold values are listed in Supplementary Tab. S2.

**Figure 12:**
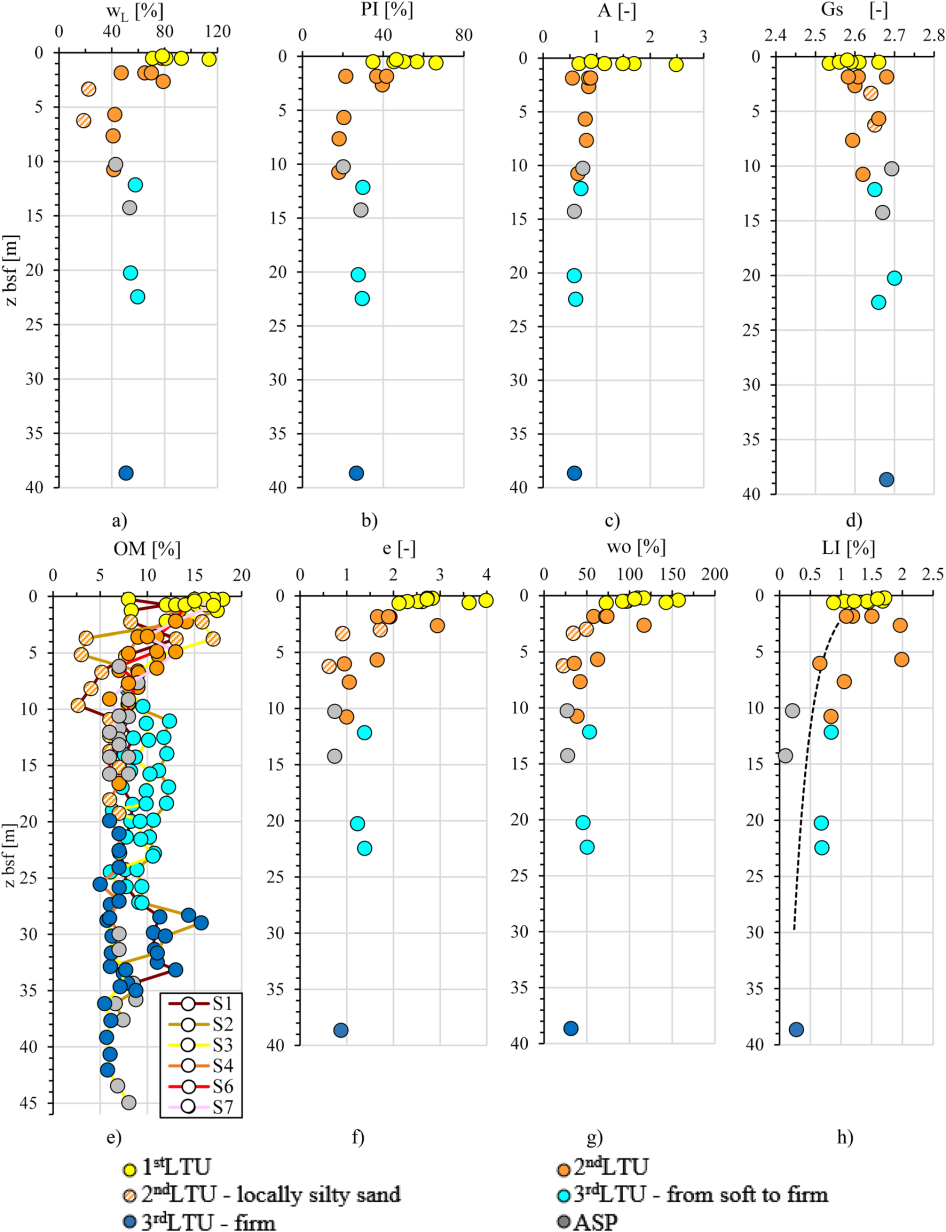
Profiles of chemo-mechanical properties of the samples collected within *LTU*s (S1, S2, S3, S4, S6, S7 close to section N–N’ in Fig. 2): (**a**) liquid limit, w_L_; (**b**) plasticity index, PI; (**c**) activity index, A; (**d**) soil solid specific gravity, Gs; (**e**) organic matter, OM; (**f**) void ratio, e; (**g**) water content, w_0_; (**h**) liquidity index, LI.

For some of the geotechnical properties, Fig. 12 reports the profiles grouped together in a single plot, as well as a plot of all the OM content profiles. It is evident the ubiquitous presence in the section of a top layer, up to 2 m thick, which roughly corresponds to the 1st *LTU*, where the sediments are of highest liquid limit (w_L_ = 70–113%), plasticity index (PI = 35–66%) and activity index (A up to 2.5), and lowest soil specific gravity (G_s_ = 2.54–2.66), much lower than that typical for the clay minerals forming the skeleton of the sediments within this layer (e.g. G_s_ = 2.75–2.78 for montmorillonite, G_s_ = 2.74 for illite and G_s_ = 2.62–2.66 for kaolinite)^94^. The low specific gravity is recognisably due, at least in part, to the major OM content in this layer, 15–18% (Fig. 14e), much higher than that usually measured in the sediments at the sea floor of open marine basins^56^. In addition, the soils in this top layer have the highest void ratio, e_0_ = 2.12–3.98 (Fig. 12f) and water content, w_0_ = 72–157% (Fig. 12g), even higher than the corresponding liquid limit (w_L_). Hence, their liquidity index LI:

1 $$LI=\frac{{w}_{0}-{w}_{P}}{{w}_{L}-{w}_{P}}= \frac{{w}_{0}-{w}_{P}}{PI}$$

is higher than 1 (Fig. 12h), as confirmed by the very low S_u_ values (Fig. 11). Therefore, the combined analysis of the data in Fig. 11 demonstrates that the very soft 1st *LTU* (S_u_ < 20 kPa), in the interface between the sediment deposit and the seawater column has geotechnical properties highly affected by the high OM content and, possibly, by the presence of the significant contaminant concentrations discussed in the following and leads to suppose that it is highly prone to remixing and resuspension.

Within the 1st *LTU,* only at site S1 the shallowest sample, which is of higher sand fraction (SF = 29.4%), has lower organic content (OM = 8–12% in Fig. 12e) than all of the other shallow samples in the section. This is likely to be due to the hydrodynamic conditions of the channel area where the site is located^95,96^. In addition, the ratio of organic carbon to organic nitrogen, C_org_/N_tot_, measured in the I Bay in previous studies^24,97^, reaches very high values close to both the sampling sites S1 and S2 (15 < C_org_/N_tot_ < 65), suggesting that the organic matter in the channel area is largely allochthonous^98^.

**Figure 13:**
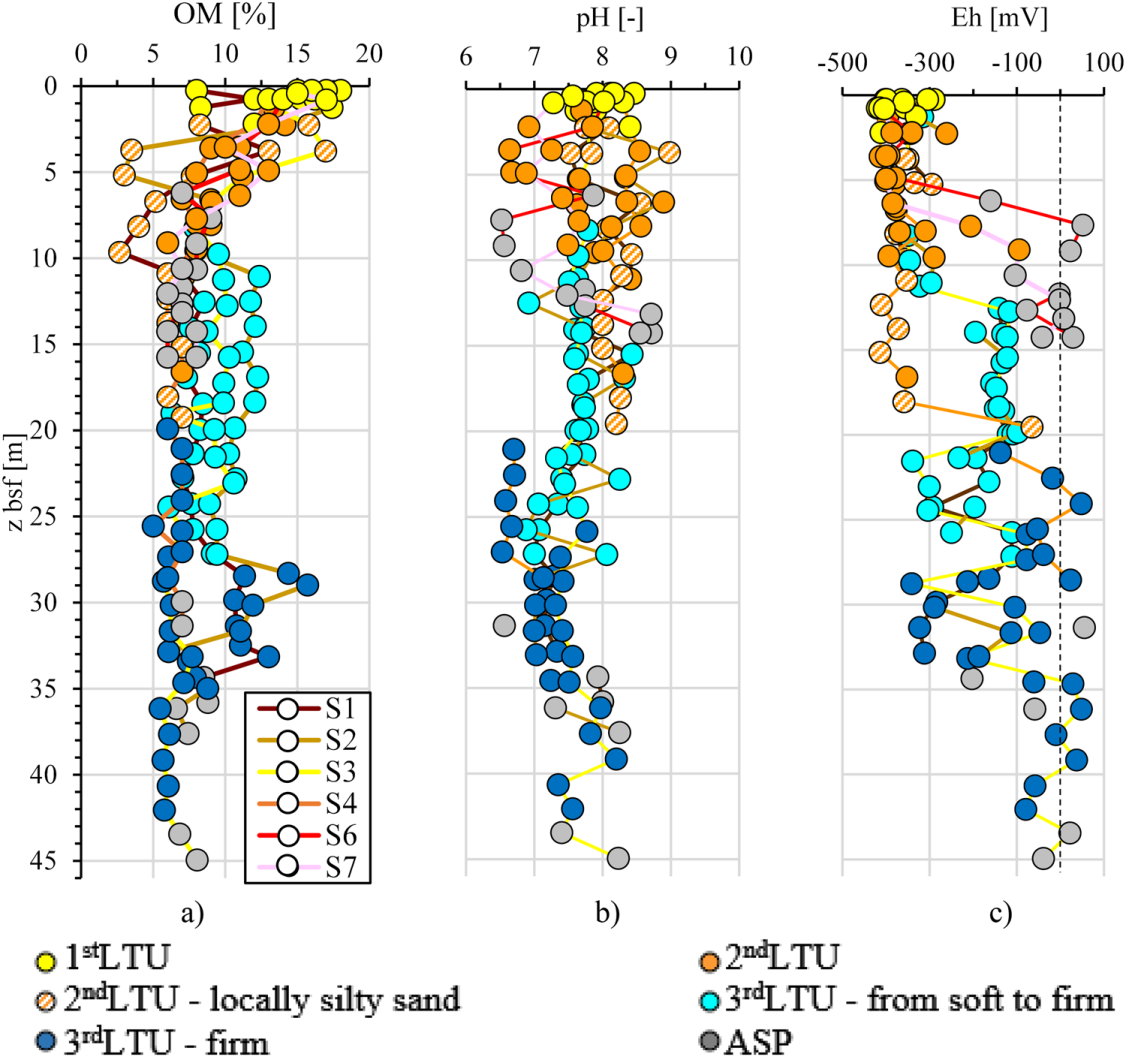
Profiles of physical properties of the samples collected at the B boreholes from different *LTUs* (sites S1, S2, S3, S4, S6, S7 close to section N–N′ in Fig. 2): (**a**) organic matter OM; (**b**) pH; (**c**) redox potential Eh.

As to the physical–chemical properties, the sediments within the 1st *LTU* are characterised by neutral to slightly alkaline conditions, with pH values in the range 7.3–8.3 (Fig. 13b), and Eh in the range from − 400 to − 200 mV (Fig. 13c), indicative of a far more reducing environment with respect to that at the sea floor in both the Adriatic and the Ionian Sea, where positive Eh values are measured^56^. The negative redox potential reveals a high rate of oxygen consumption in the I Bay, even before sediment deposition, which is likely to be due to aerobic microbial-mediated redox-processes, that can reduce the redox potential over − 300 mV and cause the total depletion of oxygen, used as terminal electron acceptor. After deposition, anaerobic mediated redox-processes are activated, such as anaerobic sulphate reduction (SO_4_^2−^ to S^2−^) or anaerobic methanogenesis, this latter converting the CO_2_ produced by the mineralisation of the organic matter, into CH_4_.

The chemical data (Supplementary Table S1) plotted in Fig. 11 reveal that in the *1*^*st*^*LTU*, down to 1.5 m depth, some of the contaminants, either organic or inorganic, exceed either the red or the yellow thresholds cited above. In 4 of the 6 sampling sites along the section, S2, S3, S4 and S6, the concentration of metals Hg, As, Pb, Cu and Zn, either approach or exceed the Taranto Site (yellow) threshold (Fig. 11, Supplementary Table S2). In addition, values of Hg above the National Environmental Law (red) threshold are recorded at both sites S3 and S6, in the front of the Navy area. In particular, the highest concentrations of the cited metals measured in the samples taken in the first 0.5 m depth are: Hg = 15 ppm at S6, Pb = 262 ppm at S3, Cu = 88 ppm at S6, Zn = 403 ppm at S6, As = 45 ppm at S6, Cd = 1.16 ppm at S6 (Supplementary Tab. S1).

**Figure 14:**
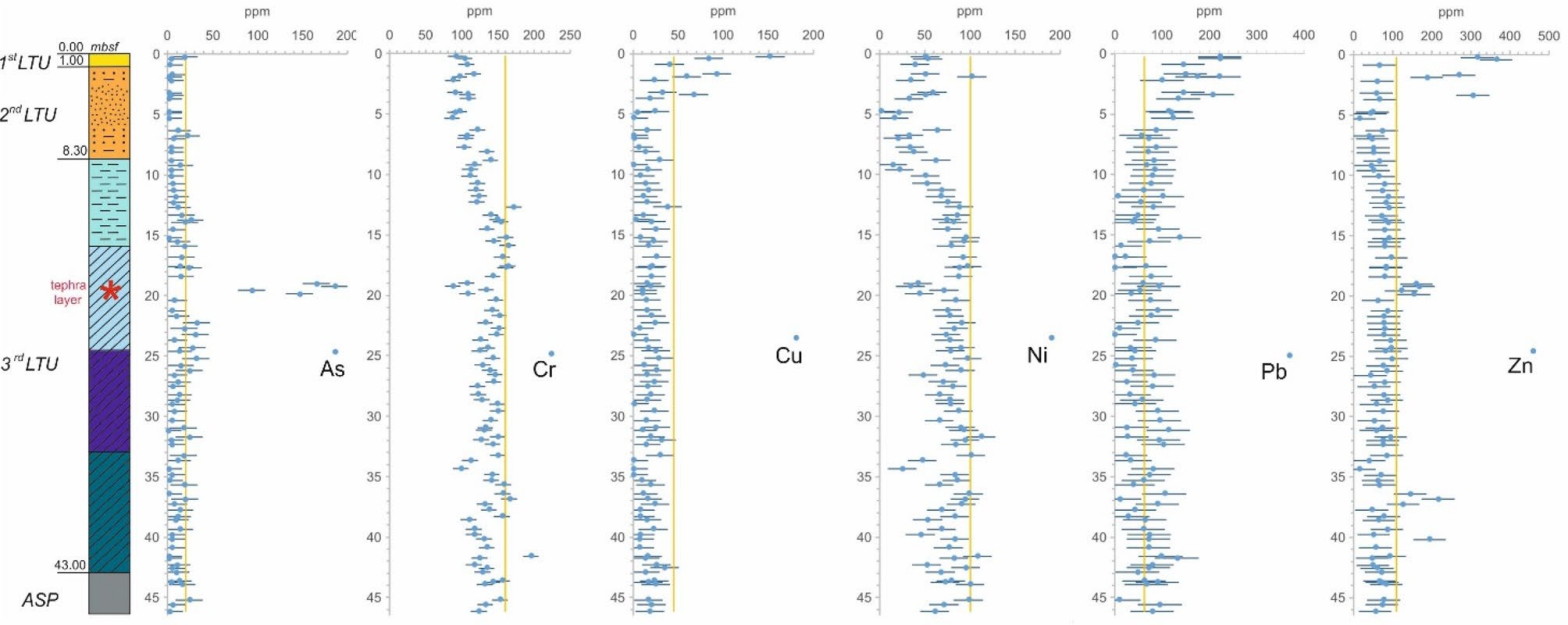
PXRF chemical profiles acquired through the scanning of the cores from S3. For each data, the error bar is also shown. The yellow line represents the Taranto Site threshold^53^. Note the geochemical anomalies at 19–21 m depth, corresponding to a tephra layer.

High metal concentrations are also recorded between 0.5 m and 1.5 m depth, although lower than in the top 0.5 m depth (Fig. 11). Unlike the other metals, the concentrations of Cr, Ni and V are well below the Taranto Site (yellow) threshold (Fig. 11) in the whole 1st *LTU*. The highest concentrations of As, Pb, Zn and Cu were recorded to occur within the top 0.5 m sediment layer of the 1st *LTU* also through the PRXF technique (Fig. 14). Since this technique detects the concentrations within spots of 6 mm diameter, very high concentrations of some metals were detected through its application also down to 4.0–5.0 m depth, as shown in Fig. 14.

Within the first 0.5 m stratum of the 1st *LTU*, the highest concentrations of the organic contaminants were recorded too (e.g. the overall sum of either the congeners of PCBs, or the congeners of PAHs), found to exceed even the National Environmental Law (red) threshold at several sites (Fig. 11). In particular, the highest values were recorded at S2 for ΣPAH_16EPA_ (9775 ppb), at S6 for ΣPCB_31_ (6828 ppb), and at sites S2, S4, S6, S3 for TPH (750 ppm; Fig. 11). The concentration of the organic contaminants reduces below 0.5 m depth.

In the 2nd *LTU* and 3rd *LTU,* both the geotechnical indices PI and A reduce with increasing depth (Figs. 11 and 12), tending to the values typical for the Pleistocene *ASP* clays^33,41,99,100^*.* Also the void ratio and the liquidity index decrease, due to both the compression of the sediments under burial and the reduction in content of either the OM or the contaminants.

**Figure 15:**
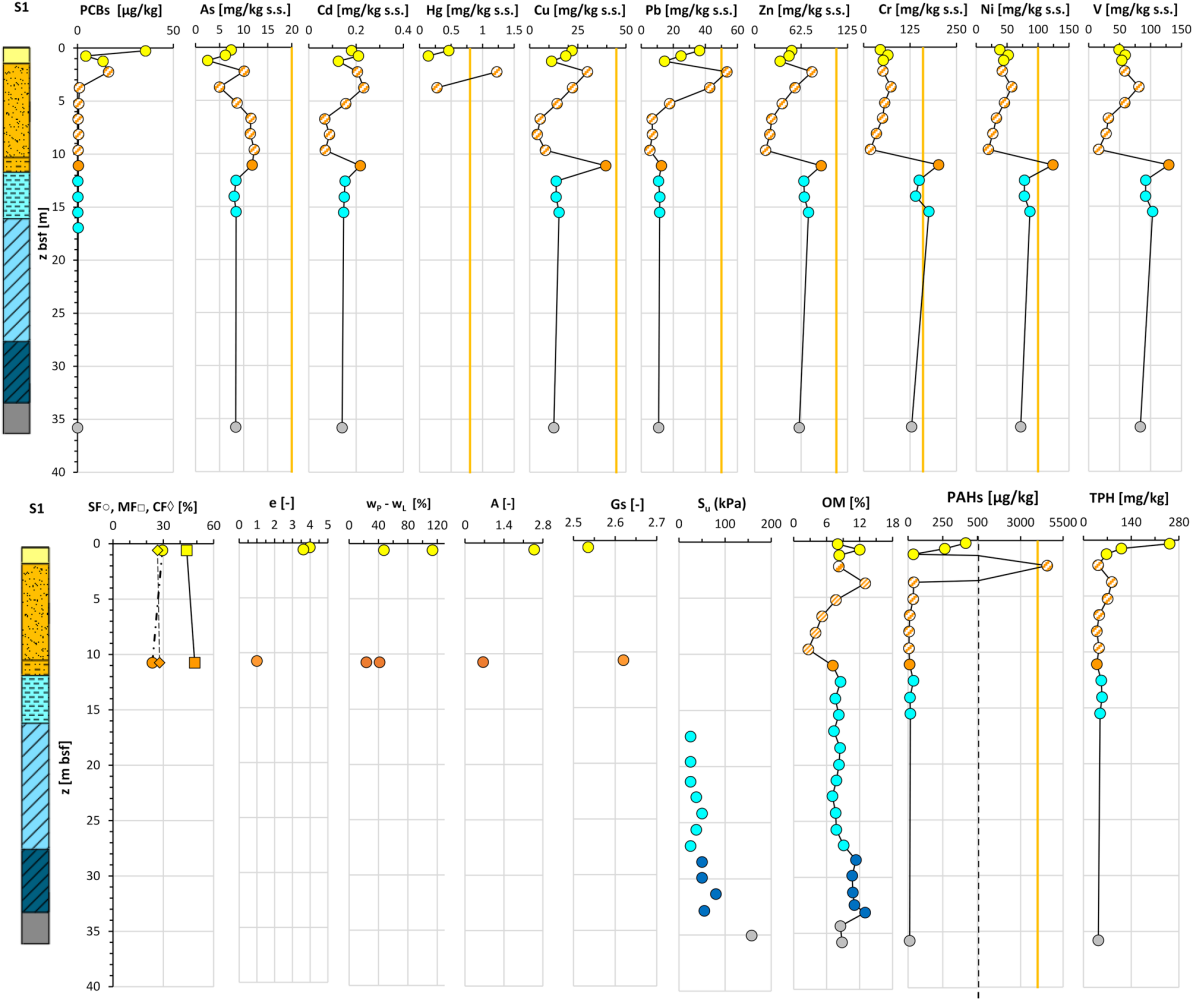
Vertical profiles of chemical and geotechnical properties along the S1 borehole in the I Bay of the Mar Piccolo site.

However, in the 2nd *LTU* the soil void ratio, the liquidity index and he OM content of the fine sediments may be still quite high by 5 m depth (Figs. 11 and 12). As expected^97,101^, a lower OM content is recorded in the sandy layer in the western part of the section (OM = 2.5%), which is, therefore, less capable to trap the contaminants, with respect to the surrounding finer soils. As to the physicochemical properties, in the 2nd *LTU* the pH values (Fig. 15b) change among the different sites, although they keep being from neutral and slightly alkaline in the eastern sites, S6 and S7, and tend to increase in the sandy layer intercepted in the western sites. The negative Eh values (Fig. 13c) of the 2nd *LTU* suggest that the process of degradation of the organic matter occurs under anaerobic conditions in both the first two units.

Contamination is heterogeneous within the 2nd *LTU*. A significant decrease in concentration of the metals with respect to the 1st *LTU* is detected, except for few local high values. For example, at about 2.25 m depth at sites S1, S2 and S3, Hg concentrations above the Taranto Site threshold (yellow) have been recorded. In addition, concentrations exceeding the Taranto Site threshold are recorded for: Zn at 2.5 m depth at site S3; Pb, down to 4 m depth at both sites S1 and S3; Cu, at 6 m depth at site S2. Furthermore, high concentrations of PAHs and PCBs have been recorded at sites S1 and S3, even exceeding the Taranto Site threshold (e.g. ΣPAH_16EPA_ ≅ 4592 ppb at 2.25 m b.s.f. in S1; ΣPCB_31_ > 190 ppb at 3.55 m b.s.f. in S6). In addition, in S3, at 2.25 m b.s.f. the concentration of TPH (> 750 ppm) exceeds the National Environmental Law threshold.

The 3rd *LTU*, from 10 to 40 m b.s.f. (Figs. 7a and 11), is characterised by a low variability of the geotechnical index properties indicative of the soil composition (Fig. 11; average values: w_LAV_ = 50%, PI_AV_ = 28%, A_AV_ = 0.65 and G_sAV_ = 2.67), which are all very close to the values characterizing the *ASP* clays. The void ratio reduces with increasing depth (e_0_ = 1.38–0.88), in a way compatible with the compression of the soil under burial (LI < 1; Fig. 12h). However, the OM content recorded in the soft to firm portion of the 3rd *LTU* (i.e. down to 35 m depth; light blue in Fig. 12e) is still quite high (OM = 6–12%), whereas it reduces to 5–6% only in the deepest firm portion (blue in Fig. 12e). Therefore, on the whole, the OM profile (Fig. 12e) suggests that the decomposition of the organic matter buried within the 3rd *LTU* has been anaerobic and so slow as to preserve high OM contents at depth long time after deposition. Such hypothesis is validated by the measured values of the redox potential and the pH in this unit, which are still typical of a reducing environment in large part of the unit (− 100 mV; Fig. 13), and tend to zero, or to positive values, only when the sediment becomes firm at large depth (blue in Fig. 13c). The high OM content of this unit justifies the G_s_ values slightly lower than those typical for the *ASP* clays (for *ASP* G_s_ = 2.73^33,41,99,100^).

In the 3rd *LTU*, though, the concentrations of the metals and of the organic contaminants are very low at all sites (Fig. 13), with the only exceptions of the lithogenic metals Cr, Ni, Zn and V, whose concentrations keep being high in most part of the unit. At sites S1 and S2, both Cr and Ni even exceed the Taranto Site threshold, from 11 m b.s.f. and 17 m b.s.f. downwards, respectively (Fig. 11). Also the PRXF profiles (Fig. 14) indicate that the concentration of either Ni or Cr increases at the depth of transition between the 2nd *LTU* and the 3rd *LTU*. They also show an excess of As, Pb and Zn down boreholes S1, S2 and S3, at about 19–21 m depth, probably relating to the presence of the thin layer of volcanic soils (tephra) recognized through the geological analyses.

Finally, the samples belonging to the *ASP* formation (Figs. 11 and 12) are characterised by values of the index properties (i.e. w_LAV=_48%, PI_AV_ = 24%, A_AV_ = 0.66, G_sAV_ = 2.71: w_0AV_ = 27%, e_AV_ = 0.74, LI_AV_ = 0.15) consistent with the average values usually measured for such clays either in land, or at depth below the Mar Grande seafloor^32,33,41,99,100,102^. The OM content of the *ASP* clays is lower than that of the overlying units (OM = 6–8%), irrespective of depth (Fig. 12e) and the redox potential becomes positive (i.e. about 50 mV, Fig. 13c). As in the 3rd *LTU*, high concentrations of Cr, Ni, and V are recorded in the *ASP* clays, which demonstrate that the content of such metals recorded in both the 3rd *LTU* and the *ASP* clays is part of the skeleton of the sediments in these units, as further discussed in the following.

## The conceptual site model

The data presented above allow for an insight into the relations between the distribution of the OM content and of the contaminants, either inorganic or organic, across the I Bay seafloor deposit, and the variability in geotechnical properties of the soils. Such insight allows for the construction of the conceptual model of the contaminated system, which represents the tool for the prediction of the fate and mobility of the contaminants and the assessment of the contamination hazard. The conceptual site model refers here to the southern part of the Mar Piccolo I Bay, but a three-dimensional conceptual model of the whole I Bay will be covered in a following paper according to the same methodology presented in this paper.

As recognized before, the variability in sediment granulometry and state parameters (w_0_, e_0_, LI) reduces with increasing depth, since the soil geotechnical properties get close to those typical for the *ASP* clays by the mid depth of the 2nd *LTU*. The OM is high in the 1st *LTU* and in the top portion of the 2nd *LTU*, but it reduces progressively with increasing depth, to become quite constant in the 3rd *LTU*. The concentrations of the organic contaminants and of several metals, such as Hg, As, Pb, Cu, Zn, are highest in the 1st *LTU* and remain locally high also in the 2nd *LTU.* Conversely, Cr, Ni and V are found to increase in both the 3rd *LTU* and the *ASP* clays.

The high levels of OM, together with the high concentration of either the organic or the inorganic contaminants in the 1st *LTU*, appear to activate coupled chemo-mechanical processes which confer to the sediments in this unit values of the geotechnical properties indicative of the soil composition which are not consistent with the soil granulometry and mineralogy. High OM content appears to correspond to higher values of w_L_, PI and A, and lower values of G_s,_ with respect to those applying to the corresponding inorganic soil (Figs. 17, 18), as already observed in the literature for other organic soils^103–105^.

The high OM content in the 1st *LTU* and in some portions of the 2nd *LTU* is likely to be partly the remain of peat brought in the basin during Holocene transgression stages, as usual in coastal environments. Otherwise, it may be effect of either the biogenic accumulation of “algal carpets” at the sea bottom^29,39^, or the long-lasting mussel farming activities carried out in the bay. The recognition that the very high w_0_ and LI values within the top sediments are effect of coupled chemo-mechanical processes is confirmed by the observation that such sediments are not of the liquid consistency expected for uncontaminated soils^106^. This is why these sediments could be sampled, irrespective of their high w_0_, and exhibited a higher cohesion, which is typically conferred to soils by high OM contents^107^. Nonetheless, as consequence of the high LI values, the 1st *LTU* sediments and those in the upper part of the 2nd *LTU* are prone to be remoulded and resuspended when impacted by the dragging of ship anchors and the navigation activities. Such finding suggests that the recorded local presence of contaminants at depths as high as 2.25–3 m may have been caused by the sediment remoulding. Furthermore, the persistence of a low consistency of the sediments down to depths higher than usually expected (e.g. 4–5 m depth), is recognized to be a finding of great interest in the design of the contamination remedial measures, since it poses additional challenges to the success of either standard capping measures, or traditional dredging activities.

The measured values of Eh and pH within both the 1st *LTU* and the 2nd *LTU* are typical of a reducing environment and suggest that the organic matter buried during the building up of such units has undergone a slow anaerobic degradation, leaving significant part of the buried OM only partially degraded. This is why the OM keeps being relatively high down to medium–large depths. It is worth recalling that the fate of the OM in the sediments impacts the fate of the contaminants^101,108^, since the degradation of the OM may trigger leaching phenomena and the concurrent release of the contaminants from the sediments to the water column.

**Figure 16:**
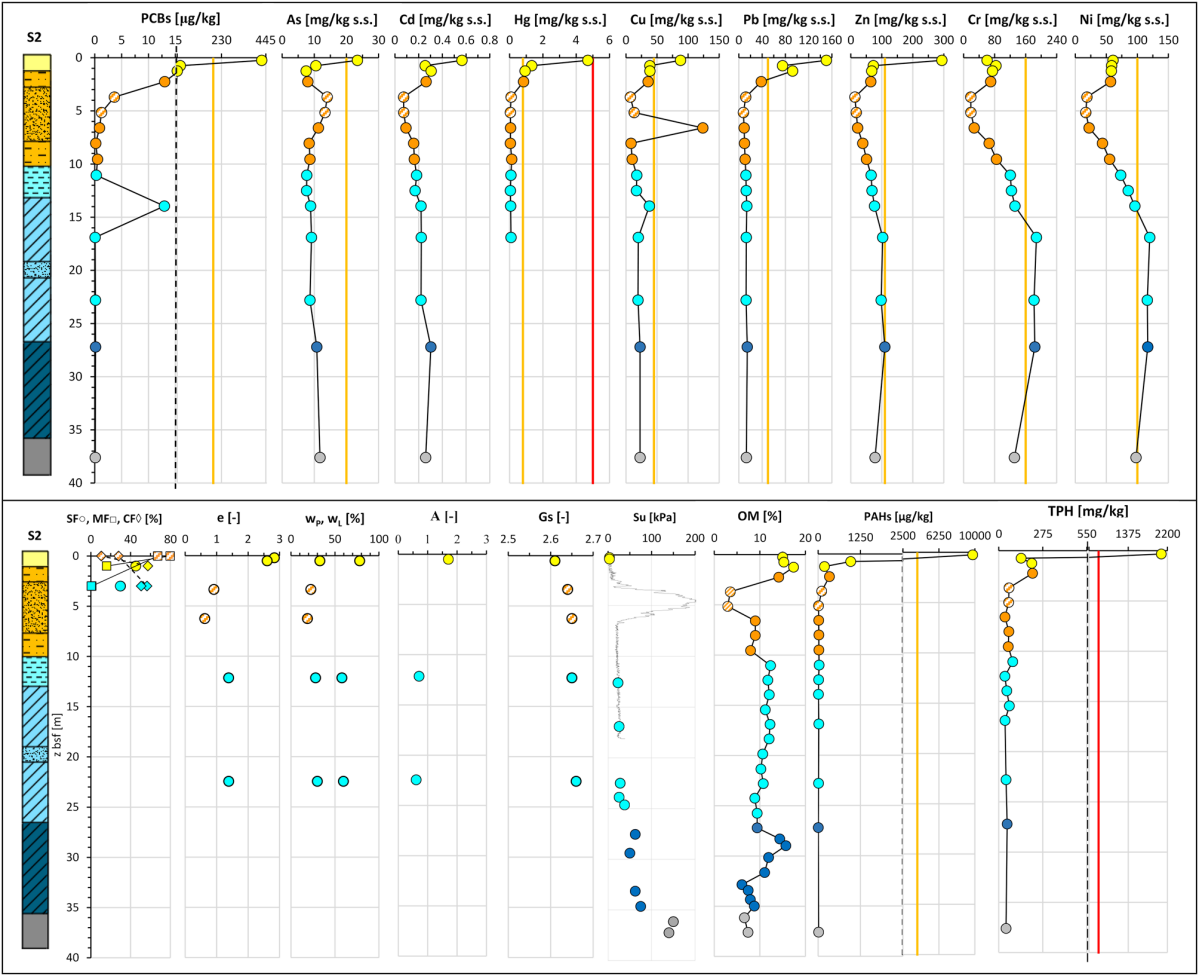
Vertical profiles of chemical and geotechnical properties along the S2 borehole in the I Bay of the Mar Piccolo site.

**Figure 17:**
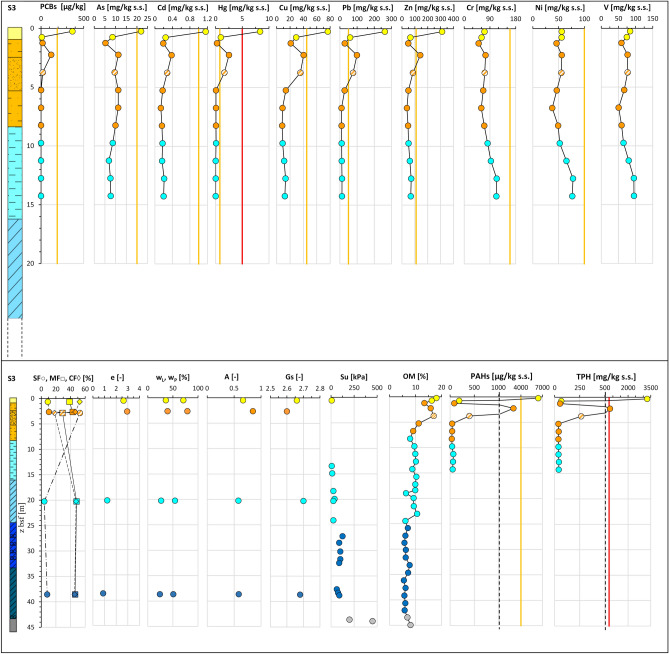
Vertical profiles of chemical and geotechnical properties along the S3 borehole in the I Bay of the Mar Piccolo site.

**Figure 18:**
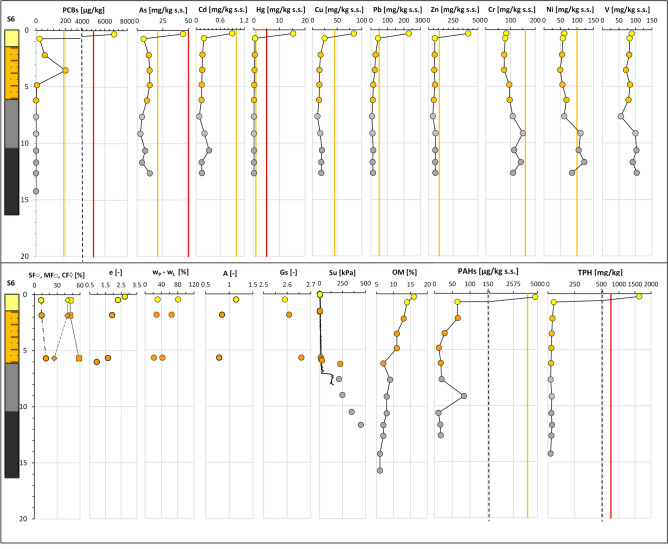
Vertical profiles of chemical and geotechnical properties along the S6 borehole in the I Bay of the Mar Piccolo site.

Figures 15, 16, 17, 18 reveal also the influence of the sediment granulometry on the sediment capacity to trap contaminants^18,20,95,96,101,108–112^. In particular, the transition from the fine sediments to the coarser ones (more sandy) in in the western part of the 2nd *LTU* (sites S1 and S2 in the N–N′ section) is clearly characterized not only by an abrupt variation in the sediment geotechnical properties, but also by a reduction in concentration of all the contaminants and in OM content. However, it is worth recalling that in addition to granulometry, also the sediment textural features and mineralogy influence the quantity and the state of the OM and of the contaminants in soils. This is because the clay minerals exhibit a high capacity of contaminant adsorption, through short-range chemical forces, such as ionic or covalent bonding^109,112^. Furthermore, the finest sediments hinder the diffusion of oxygen at depth, thus favour the preservation of the organic matter^101^ and, consequently, the absorption of organic particles onto the charged surfaces of the clay minerals. On the other hand, such preserved OM, trapped in the clayey sediment, owns terminal charges OH^−^ and COO^−^, which can further either complex or adsorb contaminants (insoluble inorganic particles and organic colloids). As a consequence, the combination between the capacity of the clay minerals in the fine sediments to retain contaminants, and that of the significant OM trapped by the clay particles to absorb/complex contaminants, makes the I Bay seafloor sediments prone to accumulate contaminants^18,97,110,113^. This is confirmed by the high concentration of contaminants found in the *1*^*st*^*LTU,* when either OM or CF increases*,* and by the depletion of contamination recorded, instead, within the sand stratum in the 2nd *LTU* (SF = 80%).

Needs to be mentioned that just above the sand stratum in the 2nd *LTU* (Sites S1 and S3), a local peak in contaminant concentration is recorded (2.25–3 m depth), where the concentrations of As, Cd, Cu, PCBs come close to the Taranto Site threshold, whereas Hg, Pb, Zn and PAHs (in terms of ∑PAH_16EPA_), even exceed such threshold and TPH exceeds the National Environmental Law threshold. This peak suggests that mixing processes take place in the low consistency sediments of the *1*^*st*^*LTU*, causing the contamination of the top of the sand level. The occurrence of such mixing represents one of the main causes of the contaminant mobility in the basin, which is here shown to be possibly directed not only towards the water column, but also towards the deeper sediments. This aspect of the site conceptual model is of major impact in the selection of the mitigation strategies, since it implies that the contaminated soil volumes in the basin are larger than the very top 0.5–1 m depth stratum, and may evolve with time, depending on the external actions.

At larger depths, within the fine soils of either the 3rd *LTU* or the *ASP,* the concentrations of Cr, Ni, V and Zn are found to be high, even exceeding the Taranto Site threshold (Figs. 11  and  15, 16, 17, 18). Such high concentrations may be justified by the geological origin of the deep sediments, since they derive from the erosion of the *ASP* clays outcropping inland, whose mineralogy has been discussed in “The Litho-technical characterization of the deposit” section. In particular, for the main clay minerals present in these clays, Zn, Ni, V, Cr are generally present in the crystal structure^114^. As such, they are found in the 3rd *LTU*, which has mainly resulted from the sedimentation of such eroded soils in the late Pleistocene and early Holocene. Therefore, the Taranto Site thresholds^53^ for Zn, Ni, Cr should be revised in light of the geological origin of the sediments, i.e. of the site-specific geochemical background. Furthermore, since Zn, Ni, Cr and V are part of the soil skeleton, they are characterised by a lower degree of mobility than the other metals and should not be then considered priority targets in the remediation strategies. It is interesting to notice that, at large depth, also the concentration of Cu and, to a less extent, Cd, follow variations similar to those of Ni, V and Cr, suggesting that, in this case, also these two metals might be of lithogenic origin.

The remaining metals, As, Hg, Cd, Pb, and Zn for the top unit, follow a spatial variation pattern which is, in first approximation, similar to that applying to the organic contaminants PCBs, PAHs, TPH; therefore, all these either inorganic or organic contaminants represent priority targets for the remediation strategies^115^. Their concentrations reduce significantly by 2.25–3 m depth. The similarity between the contamination patterns of the metals and of the organic contaminants suggests that they comply with a similar history of deposition, most likely triggered by anthropic activities.

The excessive concentration of PAHs by 3 m depth suggests the prevalent anthropic origin of such hydrocarbons. The molecular ratio among the different PAH congeners and isomers needs to be assessed for an insight into their origin, which may be either petrogenic (fuel loss), or pyrogenic (fuel combustions), or diagenetic (natural^111^). On the whole, the concurrence of high concentrations of Pb, PAHs and TPH, according to the contaminant profiles in Figs. 13 and 17, 18, suggests that lead-enriched fuel losses may have largely caused the contamination of the sediments, given the intense ship’s navigation in front of the Navy area. In addition, organo-metallic antifouling residues may have been the source of the high concentrations of Cu, Zn, Cd, probably used for ship maintenance in the near shipyard areas^116^.

The concentration of PCB is high not only in the 1st *LTU*, but also in local spots down to 2–3 m depth. Given the anthropogenic origin of such contaminants their local presence at such depth may be due to the remixing of the top sediments, where usually these contaminants are found. Finally, it is worth highlighting the low contaminant concentrations recorded at both sites S1 and S7, located close to channel areas. At site S1, the contaminant concentrations in the top layer of the *1*^*st*^* LTU* resulted to be unexpectedly lower than those recorded in the deeper sediment envisaged that the peculiar hydrodynamic conditions applying to the areas where these sites are located impact the distribution of the contaminants, since the finest sediments of the 1st *LTU* are prone to be resuspended and transported by the marine currents^95,96^.

## Conclusions

The multidisciplinary study presented in the paper has proven to be successful in achieving an insight into the distribution of the contaminants in the southern part of the Mar Piccolo I Bay, as well as into their origin, mobility and fate, which provide rational indications about the hazard of the site. Such insight has been pursued thanks to the innovative aspects of the study with respect to traditional characterization approach of the polluted sites and consists in the tight collaboration among researchers of different fields in a join interdisciplinary analysis of the investigation results, as well as the deterministic assessment of the pollution hazard as the main target of the innovative campaign. Given such target, the investigation and testing have been designed to measure, aside the contaminant concentrations, all the data characterizing the factors influencing the contaminant mobility and their fate within the seafloor deposit, essentials in providing knowledge about the current state and the evolution with time of the site pollution. In order to tackle the challenge of characterizing all these factors, the seabed of the site has been explored down to large depths and the boundary conditions of all the chemical and hydro-mechanical processes that may impact the contaminant mobility have been deeply analyzed.

It has been shown that in the southern portion of the I Bay, the concentrations of either organic or inorganic contaminants reach the highest level within the fine sediments of the very shallow layers, that resulted to be the most exposed to the anthropogenic contaminations. Nevertheless significant concentrations are locally recorded at larger depths. The fine sediments that are results of the inland erosion of marine formations and illitic clays (*ASP*), tend to trap the contaminants not only for their high clay fraction, but also for the significant content of organic matter, revealing that the coupling phenomena between textural features and the cycle of the organic matter within sediment control the distribution of pollutants and their fate. The multidisciplinary research study has demonstrated also that in some cases (i.e. Zn, Ni and Cr), the background contaminant thresholds need to be revised in light of the site-specific geochemical variability of metals having lithogenic origin.

Furthermore, the OM resulted poorly degraded down to few metres depth and, as such, confers to the sediments much higher plasticity and cohesion than expected according to their composition. Consequently, the sediments are fluid at the sediment–water interface, and of very low consistency below, even when buried at few metres, as effect of chemo-mechanical coupled phenomena. It follows that, being prone to undergo remoulding and re-suspension due to external actions, they represent by themselves a predisposing factor for the spatial and vertical migration of pollutants, processes that need to be taken into account in the selection of the proper remediation strategy.

The investigation reveals also that the mobility of the contaminants as effect of an advective upward flow from the deep karst artesian aquifer is likely to be minor, due to the large thickness of sediments of very low permeability covering the artesian aquifer, which is characterized by a piezometric level of 1 m above sea level. Therefore, the hydrogeologic regime of this portion of the bay is not expected to contribute to the site contamination hazard. However, further studies are ongoing to numerical modelling the hydro-dynamic dispersion of the contaminants, by using as input data all the chemo-hydro-mechanical parameters collected in the present investigation. The numerical simulations are entailing also the loading and unloading processes at the base of traditional remedial strategies, like capping or dredging, and account for the ultra-soft consistency of the top sediments that makes such interventions peculiarly challenging.

The several insights into the features and possible sources of the contaminants in the system under study confirm the efficiency of the cooperative approach promoted by the Special Commissioner, that can be largely used by decision makers as premise of the selection of the most sustainable mitigation measures and as support to the risk management process.

## Supplementary Information

Supplementary Information.

## Data Availability

The datasets generated during the current study are not publicly available and are used through a specific clearance issued by the Special Commissioner for urgent measures of reclamation, environmental improvements and redevelopment of Taranto. The data are available at the reasonable request and with permission of the Special Commissioner for urgent measurements of reclamation, environmental improvements and redevelopment of Taranto.
