# Supplementary material for: A geo-chemo-mechanical study of a highly polluted marine system (Taranto, Italy) for the enhancement of the conceptual site model
Source: Sci Rep. 2021 Feb 17;11:4017. doi: 10.1038/s41598-021-82879-w (PMC7889905; doi:10.1038/s41598-021-82879-w)
Supplement: Supplementary file 1 — Supplementary Information. [file 41598_2021_82879_MOESM1_ESM.doc]

**Supplementary Information**

**A geo-chemo-mechanical study of a highly polluted marine system (Taranto, Italy) for the enhancement of the conceptual site model**

Cotecchia F.^a1^, Vitone C.^a1^, Sollecito F.^a1^**^*^**, Mali M. ^a1^**^*^**, Miccoli D.^a1^, Petti R.^a1^, Milella D.^a1^, Ruggieri G.^a1^, Bottiglieri O.^a1^, Santaloia F.^b^, De Bellis P.^a1^, Cafaro F.^a1^ , Notarnicola M.^a1^, Todaro F.^a1^, Adamo F.^a2^, Di Nisio A.^a2^, Lanzolla A.M.L. ^a2^, Spadavecchia M. ^a2^, Moretti M. ^c^, Agrosì G. ^c^, De Giosa F. ^c^, Fago P. ^c^, Lacalamita M. ^c^, Lisco S. ^c^, Manzari P. ^c^, Mesto E.^c^*,* Romano G.^c^, Scardino G.^c^, Schingaro E.^c^, Siniscalchi A.^c^, Tempesta G.^c^, Valenzano E.^c^, Mastronuzzi G.^c^, Cardellicchio N.^d1^, Di Leo A.^d1^, Spada L.^d1^, Giandomenico S.^d1^, Calò M.^d1^, Uricchio V.F.^d2^  Mascolo G.^d2^, Bagnuolo G.^d2^, Ciannarella R.^d2^, Tursi A.^e^, Cipriano G. ^e^, Cotugno P.^e^, Sion L.^e^, Carlucci R.^e^, Capasso G.^f^, De Chiara G.^f^, Pisciotta G.^f^, Velardo R ^f^., Corbelli V^f^.

^a1^ DICATECh – Department of Civil, Environmental, Land, Building Engineering and Chemistry, Polytechnic University of Bari - via Orabona 4, 70125 Bari, Italy

^a2^ DEI – Department of Electrical and Computer Science Engineering, Polytechnic University of Bari - via Orabona 4, 70125 Bari, Italy

^b^ CNR - IRPI - National Research Center - Via Amendola 122/I, 70126 Bari

^c^ DISTEGEO *-* Department of Earth and Geoenvironmental Sciences, University of Bari Aldo Moro, Via Orabona 4, 70125 Bari, Italy

^d1^ CNR-IRSA *-* National Research Center - Water Research Institute, Via Roma 3, 74123 Taranto, Italy

^d2^ CNR-IRSA *-* National Research Center - Water Research Institute,Via F. De Blasio 5, 70132 Bari, Italy

^e^ Department of Biology - University of Bari, Via Orabona, 4, 70125 BARI (Italy)

^f^ Special Commissioner for urgent measures of reclamation, environmental improvements and redevelopment of Taranto

*^*^* *Corresponding author e-mail address: francesca.sollecito@poliba.it (Sollecito F.) and matilda.mali@poliba.it (Mali M.); tel:+39 080 5963338/666;fax: +39080 5963414*

#### **Supplementary Figure S1.** Biocenosis within the Mar Piccolo I Bay. Key. Grey and dark grey: seabed without algae and with scarce presence of algae respectively; orange: seabed covered by macroalgae; green: seabed covered by several species of algae (e.g. Cymodocea nodosa); blue: seabed covered by debris formed of shell fragments; black lines: border the mussel farming areas; blue circles: the main submarine springs (1 Citro Galeso, 2 Citro Citrello).


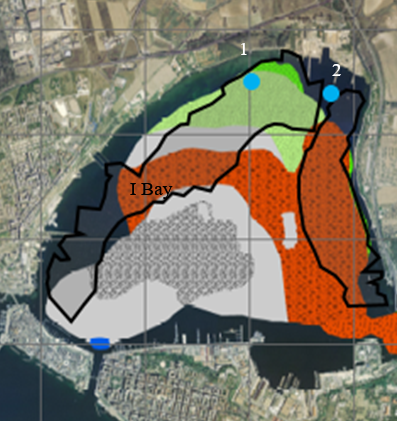


####

#### **Supplementary Figure S2.** Investigation Campaign 2017: a) The offshore platform, b) one of the polycarbonate tube samplers used within the first 1.5 m sediment stratum at the sea floor, c) one of the Osterberg tube samplers used for deeper sampling.

| 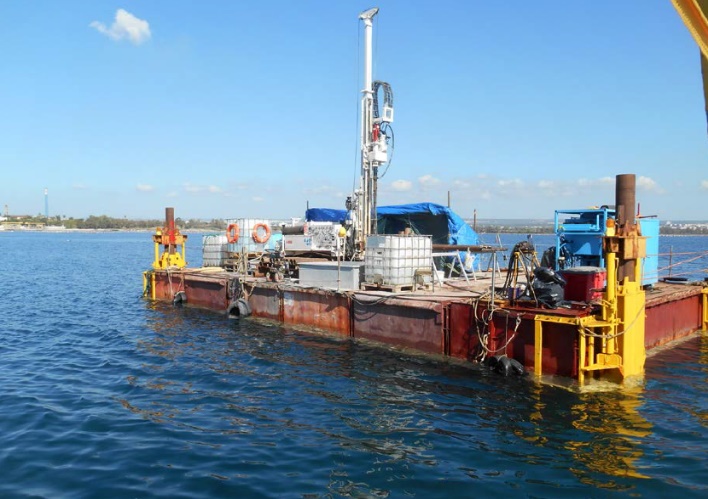 | 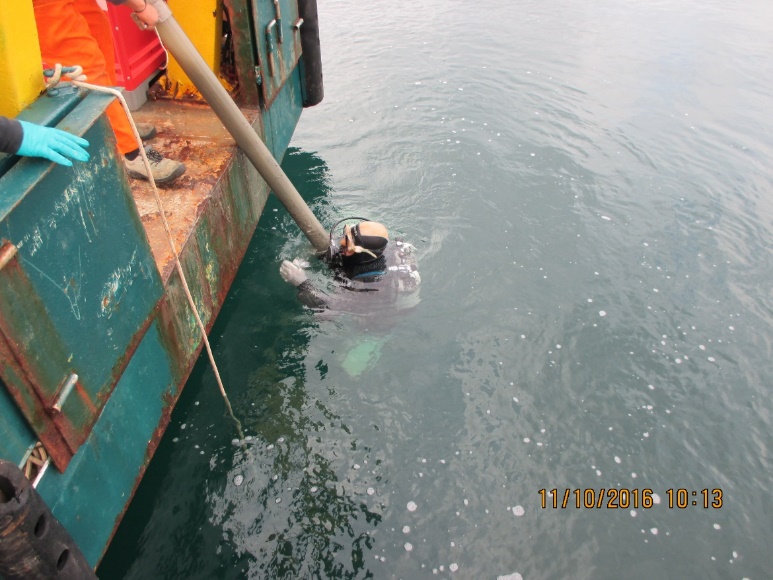 | 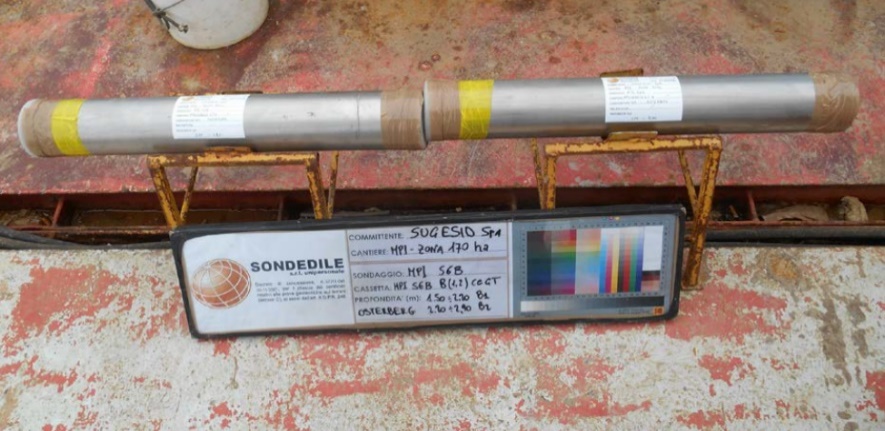 |
| --- | --- | --- |
| a) | b) | c) |

#### **Supplementary Figure S3**. (a) Representation of the boreholes (Bor.) A, B and manually retrieved, M, for each site; for borehole B also the sub-sampling is shown, for geotechnical (1), geological/geochemical (2) and chemical (3) testing. (b) Sub-sampling design for all the investigation sites. (c) Data of the investigation campaign carried out by the Special Commissioner in the Mar Piccolo I Bay.

| 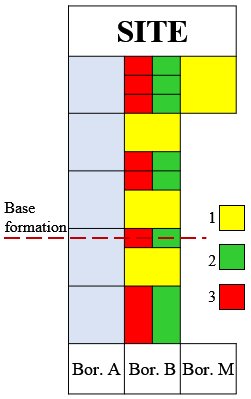 |  |
| --- | --- |
| a) |
| 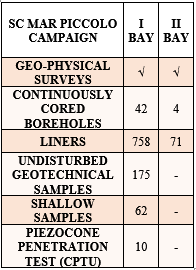 |
| c) | b) |

#### **Supplementary Figure S4**. XRPD pattern of the S2 borehole sediments. The S2BG2, S2BN2 and S2BR3 samples have been taken at 9.55, 20.17 and 26.37 m., respectively. Legend: I+I/S = Illite + Illite/Smectite; Cl: Chlorite; Kln: Kaolinite; C: Calcite; Ar: Aragonite; Dol: Dolomite; Qrz: Quartz; Plg: Plagioclase; Feld: Feldspar; Hem: Hematite; Py: Pyrite; Hal: Halite; Rut: Rutile.


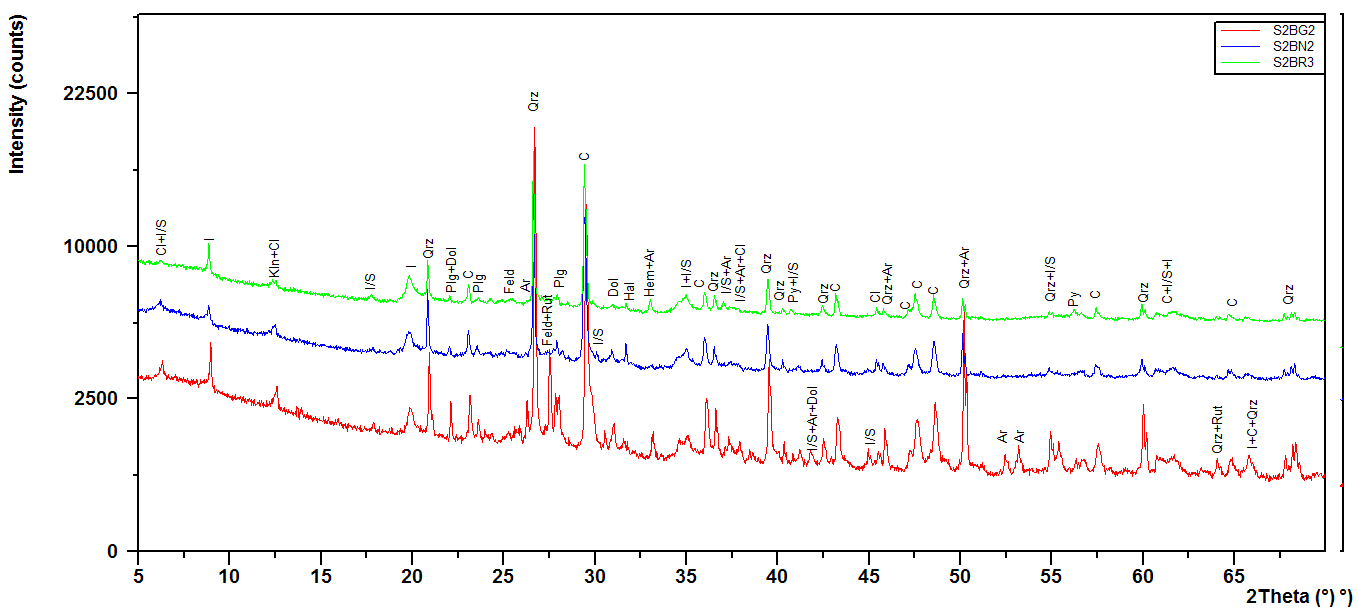


#### **Supplementary Table S1.** Contaminant Concentrations

|  | Depth | Hg | Cd | Pb | As | Cr | Cu | Ni | Zn | Fe | Mn | Sn | Al | V | ∑PCB_31_ | PAHs _∑16-EPA_ | TPH |
| --- | --- | --- | --- | --- | --- | --- | --- | --- | --- | --- | --- | --- | --- | --- | --- | --- | --- |
|  | m | mg/kg | mg/kg | mg/kg | mg/kg | mg/kg | mg/kg | mg/kg | mg/kg | mg/kg | mg/kg | mg/kg | mg/kg | mg/kg | µg/kg | µg/kg | mg/kg |
| S1 | 0.25 | 0.46 | 0.18 | 36.38 | 7.40 | 44.96 | 21.97 | 38.31 | 49.89 | 11857 | 297 | 2.58 | 26353 | 48.94 | 35.51 | 415.08 | 252.68 |
|  | 0.75 | 0.14 | 0.21 | 24.78 | 6.19 | 65.54 | 18.71 | 51.78 | 46.27 | 18206 | 353 | 1.83 | 32349 | 59.02 | 4.36 | 264.53 | 111.37 |
|  | 1.25 | - | 0.13 | 14.52 | 2.50 | 52.89 | 11.33 | 44.43 | 34.26 | 14546 | 286 | 1.21 | 27593 | 53.62 | 13.31 | 38.71 | 67.67 |
|  | 2.25 | 1.22 | 0.21 | 53.48 | 10.10 | 51.94 | 30.01 | 42.12 | 77.22 | 17348 | 376 | 4.66 | 35383 | 58.29 | 16.22 | 4562.3 | 43.08 |
|  | 3.75 | 0.28 | 0.23 | 42.73 | 4.97 | 73.52 | 22.27 | 57.65 | 54.39 | 21211 | 364 | 2.87 | 40716 | 81.08 | 1.02 | 41.53 | 83.26 |
|  | 5.25 | - | 0.16 | 17.72 | 8.64 | 56.89 | 14.22 | 45.71 | 37.18 | 15204 | 372 | 1.32 | 31616 | 58.43 | 0.65 | 37.04 | 70.98 |
|  | 6.70 | - | 0.07 | 6.81 | 11.50 | 51.06 | 5.47 | 32.74 | 23.16 | 10235 | 326 | 0.66 | 30402 | 31.87 | 0.31 | 13.08 | 46.24 |
|  | 8.15 | - | 0.09 | 6.94 | 11.39 | 34.43 | 3.92 | 27.01 | 20.43 | 8638 | 394 | 0.57 | 28431 | 28.10 | 0.54 | 8.22 | 39.34 |
|  | 9.65 | - | 0.07 | 5.39 | 12.20 | 18.34 | 8.14 | 19.81 | 14.86 | 4729 | 300 | 0.34 | 15953 | 16.01 | 0.30 | 6.88 | 45.56 |
|  | 11.10 | - | 0.22 | 12.60 | 11.77 | 201.93 | 39.51 | 124.12 | 89.60 | 48624 | 682 | 2.38 | 90473 | 129.44 | 0.47 | 11.50 | 39.35 |
|  | 12.55 | - | 0.15 | 10.78 | 8.42 | 149.50 | 13.76 | 78.12 | 66.21 | 34554 | 629 | 1.89 | 72576 | 92.19 | 0.18 | 39.52 | 52.21 |
|  | 14.05 | - | 0.15 | 11.60 | 8.02 | 140.00 | 13.71 | 77.72 | 66.91 | 34132 | 628 | 1.95 | 70422 | 91.93 | 0.20 | 13.89 | 54.47 |
|  | 15.50 | - | 0.15 | 11.50 | 8.43 | 175.86 | 15.24 | 86.82 | 72.57 | 39124 | 623 | 2.09 | 77369 | 103.35 | 0.15 | 16.72 | 48.94 |
|  | 16.95 | - | - | - | - | - | - | - | - | - | - | - | - | - | 0.34 | - | - |
|  | 35.80 | - | 0.14 | 10.86 | 8.36 | 129.23 | 12.50 | 72.05 | 60.03 | 31909 | 589 | 1.84 | 67547 | 83.45 | 0.05 | 12.41 | 43.76 |
| S2 | 0.25 | 4.70 | 0.57 | 150.38 | 23.42 | 60.45 | 87.92 | 60.83 | 293.45 | 28402 | 484 | 19.60 | 36235 | 89 | 424.82 | 9774.70 | 2071 |
|  | 0.75 | 1.33 | 0.25 | 75.06 | 10.49 | 83.28 | 38.44 | 58.66 | 72.78 | 28109 | 417 | 6.92 | 48343 | 78 | 30.08 | 962.74 | 139.56 |
|  | 1.25 | 0.93 | 0.31 | 92.39 | 7.46 | 74.28 | 38.55 | 58.01 | 67.64 | 21096 | 330 | 5.86 | 39224 | 77 | 15.70 | 188.07 | 206.22 |
|  | 2.25 | 0.84 | 0.26 | 38.47 | 8.00 | 69.80 | 35.60 | 56.88 | 64.40 | 20907 | 342 | 4.14 | 38826 | 72 | 13.02 | 335.47 | 211 |
|  | 3.70 | 0.09 | 0.07 | 11.90 | 14.01 | 18.51 | 7.11 | 18.88 | 13.34 | 5728 | 202 | 0.58 | 12922 | 17 | 3.65 | 106.41 | 63.6 |
|  | 5.15 | 0.05 | 0.07 | 7.88 | 13.34 | 18.67 | 13.15 | 16.93 | 17.69 | 5892 | 235 | 0.56 | 14121 | 19 | 1.25 | 13.15 | 61.49 |
|  | 6.60 | 0.06 | 0.09 | 9.04 | 11.27 | 27.19 | 123.77 | 22.34 | 22.10 | 7797 | 292 | 0.75 | 21972 | 23 | 0.89 | 18.40 | 38.29 |
|  | 8.05 | 0.05 | 0.16 | 9.66 | 8.41 | 65.80 | 8.12 | 43.77 | 38.59 | 18230 | 420 | 1.36 | 40922 | 49 | 0.18 | 23.61 | 62.82 |
|  | 9.55 | 0.13 | 0.16 | 10.76 | 8.67 | 84.65 | 10.05 | 55.37 | 50.83 | 23561 | 442 | 1.72 | 47714 | 66 | 0.51 | 23.06 | 58.96 |
|  | 11.05 | 0.08 | 0.18 | 12.11 | 7.65 | 120.36 | 17.39 | 73.22 | 65.59 | 35082 | 602 | 2.12 | 63148 | 90 | 0.29 | 30.78 | 86.92 |
|  | 12.50 | 0.05 | 0.17 | 12.69 | 7.60 | 123.41 | 16.97 | 85.34 | 68.36 | 32308 | 457 | 2.21 | 56720 | 100 |  | 15.20 | 37.99 |
|  | 13.95 | 0.06 | 0.22 | 13.73 | 8.85 | 132.40 | 37.71 | 95.96 | 76.60 | 38926 | 651 | 2.42 | 67236 | 111 | 12.95 | 11.94 | 49.84 |
|  | 15.45 | - | - | - | - | - | - | - | - | - | - | - | - | - | - | - | 65.62 |
|  | 16.90 | 0.09 | 0.22 | 12.82 | 9.13 | 187.66 | 19.74 | 120.02 | 102.71 | 48452 | 879 | 2.35 | 93309 | 151 | 0.07 | 20.22 | 38.77 |
|  | 22.80 | - | 0.22 | 12.43 | 8.65 | 181.58 | 19.42 | 116.10 | 97.88 | 48205 | 875 | 2.29 | 93633 | 148 | 0.13 | 13.79 | 45.13 |
|  | 27.20 | - | 0.30 | 14.43 | 10.79 | 183.65 | 22.74 | 116.73 | 109.70 | 51833 | 812 | 2.46 | 102994 | 160 | 0.15 | 5.59 | 52.25 |
|  | 37.60 | - | 0.26 | 12.98 | 11.78 | 131.02 | 22.68 | 97.94 | 78.30 | 40469 | 833 | 1.87 | 78711 | 111 | 0.10 | 15.51 | 45.08 |
| S3 | 0.25 | 8.33 | 1.16 | 261.63 | 21.91 | 71.06 | 76.61 | 56.13 | 311.43 | 24079 | 437.34 | 15.25 | 28949 | 83.81 | 366.96 | 6427 | 0.325 |
|  | 0.75 | 0.99 | 0.25 | 59.33 | 8.55 | 60.86 | 29.19 | 55.90 | 65.63 | 19159 | 435.70 | 3.90 | 29632 | 74.40 | 8.84 | 189 | 0.75 |
|  | 1.25 | 0.26 | 0.19 | 28.35 | 5.24 | 50.60 | 21.05 | 45.98 | 49.11 | 12482 | 307.57 | 2.22 | 20172 | 58.72 | 18.21 | 86 | 1.25 |
|  | 2.25 | 2.51 | 0.38 | 99.22 | 11.31 | 74.73 | 40.36 | 56.04 | 142.00 | 22848 | 430.97 | 6.03 | 31525 | 76.58 | 119.11 | 2954 | 2.25 |
|  | 3.75 | 1.65 | 0.28 | 76.78 | 9.58 | 71.83 | 35.49 | 55.36 | 85.12 | 22459 | 459.19 | 5.30 | 33739 | 76.73 | 20.74 | 400 | 3.75 |
|  | 5.25 | 0.08 | 0.18 | 27.66 | 11.25 | 66.57 | 13.75 | 46.45 | 50.02 | 21372 | 426.89 | 1.99 | 32930 | 66.84 | 0.77 | 43 | 5.25 |
|  | 6.75 | 0.07 | 0.14 | 9.50 | 11.22 | 59.69 | 8.76 | 37.65 | 38.41 | 16660 | 398.02 | 1.15 | 29125 | 50.73 | 0.50 | 43 | 6.75 |
|  | 8.25 | 0.08 | 0.16 | 10.02 | 9.94 | 70.12 | 8.19 | 49.05 | 47.52 | 15395 | 492.10 | 1.52 | 25340 | 59.12 | - | 35 | 8.25 |
|  | 9.75 | 0.10 | 0.17 | 10.36 | 8.67 | 81.94 | 8.90 | 52.23 | 51.36 | 23888 | 496.40 | 1.49 | 38494 | 64.84 | - | 42 | 9.75 |
|  | 11.25 | 0.02 | 0.16 | 11.05 | 6.88 | 93.41 | 11.16 | 65.27 | 61.33 | 27165 | 532.72 | 1.73 | 38518 | 80.00 | - | 67 | 11.25 |
|  | 12.75 | 0.03 | 0.21 | 12.43 | 7.58 | 113.63 | 13.30 | 77.92 | 71.53 | 32493 | 583.79 | 1.94 | 46735 | 95.01 | - | 60 | 12.75 |
|  | 14.25 | 0.02 | 0.20 | 12.01 | 7.72 | 112.32 | 12.22 | 76.46 | 68.48 | 33198 | 576.23 | 1.84 | 49708 | 95.27 | - | 53 | 14.25 |
| S4 | 0.325 | 4.10 | 0.63 | 129.03 | 18.51 | 90.63 | 75.17 | 74.27 | 276.63 | 34969 | 560.65 | 15.63 | 65743 | 100.15 | 1570.29 | 1361.98 | 801.00 |
|  | 0.75 | 0.76 | 0.18 | 47.53 | 7.26 | 80.81 | 34.10 | 62.00 | 69.90 | 26412 | 445.00 | 3.45 | 59327 | 78.93 | 22.01 | 87.30 | 35.00 |
|  | 2.20 | 0.05 | 0.15 | 12.02 | 10.77 | 54.93 | 10.54 | 46.17 | 42.18 | 21203 | 599.60 | 1.08 | 67743 | 50.23 | 15.68 | 19.42 | 30.28 |
|  | 3.60 | 0.08 | 0.12 | 12.54 | 11.16 | 74.25 | 9.90 | 45.74 | 50.84 | 24247 | 503.61 | 1.30 | 64972 | 58.80 | 16.06 | 14.08 | 27.31 |
|  | 5.05 | 0.08 | 0.13 | 10.86 | 10.61 | 79.45 | 8.77 | 48.88 | 56.75 | 25012 | 517.02 | 1.32 | 60901 | 62.21 | 15.84 | 15.59 | 26.03 |
|  | 6.55 | 0.13 | 0.15 | 11.86 | 8.96 | 87.11 | 8.67 | 56.16 | 55.29 | 27719 | 537.60 | 1.41 | 66385 | 65.99 | 0.10 | 14.00 | 22.85 |
|  | 8.00 | 0.06 | 0.17 | 10.79 | 6.76 | 93.13 | 10.69 | 64.94 | 57.43 | 30207 | 596.01 | 1.42 | 74860 | 70.67 | 0.47 | 11.07 | 23.70 |
|  | 9.45 | 0.05 | 0.19 | 12.74 | 7.71 | 119.51 | 14.63 | 87.97 | 73.43 | 37119 | 656.90 | 1.70 | 85938 | 91.93 | 0.50 | 42.97 | 25.72 |
|  | 10.90 | 0.06 | 0.17 | 11.29 | 8.12 | 86.14 | 11.02 | 64.54 | 51.61 | 28874 | 608.37 | 1.25 | 72420 | 66.13 | 0.45 | 33.73 | 30.70 |
|  | 12.35 | 0.06 | 0.16 | 10.79 | 7.93 | 75.61 | 9.83 | 58.32 | 47.38 | 25668 | 573.75 | 1.18 | 67187 | 60.22 | 0.61 | 14.61 | 25.72 |
|  | 13.75 | 0.11 | 0.17 | 19.98 | 13.05 | 96.39 | 15.34 | 62.88 | 75.43 | 35694 | 559.75 | 2.00 | 85321 | 83.88 | 1.44 | 15.57 | 26.78 |
|  | 15.15 | - | - | - | - | - | - | - | - | - | - | - | - | - | - | - | 26.14 |
|  | 16.60 | 0.06 | 0.18 | 10.92 | 15.77 | 76.61 | 13.03 | 62.82 | 52.55 | 29940 | 638.15 | 1.15 | 68591 | 60.40 | 0.25 | 20.46 | 36.85 |
| S6 | 0.325 | 15.36 | 0.90 | 229.29 | 44.75 | 82.85 | 83.75 | 61.70 | 403 | 25414 | 446 | 18.55 | 55449 | 88.52 | 6829.33 | 4771.56 | 1634 |
|  | 0.75 | 0.45 | 0.19 | 43.56 | 6.34 | 78.63 | 24.48 | 57.25 | 64.3 | 20824 | 389 | 2.88 | 51182 | 82.02 | 24.47 | 64.73 | 54.15 |
|  | 2.20 | 0.39 | 0.16 | 28.23 | 11.31 | 74.59 | 16.47 | 54.50 | 63.1 | 19716 | 366 | 2.50 | 45373 | 81.03 | 59.22 | 65.49 | 42.38 |
|  | 3.55 | 0.27 | 0.15 | 23.64 | 12.20 | 74.45 | 14.41 | 49.08 | 58.7 | 20897 | 387 | 1.86 | 54876 | 71.03 | 201.77 | 28.75 | 35.27 |
|  | 4.85 | 0.17 | 0.14 | 14.64 | 12.25 | 94.94 | 14.06 | 55.89 | 66.3 | 25055 | 436 | 1.62 | 67302 | 83.68 | 6.15 | 12.53 | 32.3 |
|  | 6.20 | 0.06 | 0.13 | 11.73 | 9.59 | 96.56 | 13.63 | 67.82 | 53.3 | 22463 | 416 | 1.48 | 55629 | 78.91 | 0.50 | 18.01 | 28.58 |
|  | 7.65 | 0.05 | 0.08 | 6.56 | 4.77 | 108.89 | 10.16 | 61.06 | 44.0 | 18043 | 253 | 0.89 | 43172 | 55.33 | 1.06 | 20.01 | 25.56 |
|  | 9.15 | 0.06 | 0.21 | 9.54 | 3.19 | 150.08 | 15.92 | 110.75 | 71.8 | 30931 | 399 | 1.82 | 77268 | 100.02 | 0.57 | 83.08 | 35.9 |
|  | 10.65 | 0.07 | 0.32 | 14.14 | 7.88 | 114.31 | 19.21 | 105.19 | 68.9 | 25357 | 379 | 1.89 | 60445 | 103.07 | 0.94 | 11.34 | 31.76 |
|  | 11.70 | 0.07 | 0.13 | 10.36 | 4.82 | 141.87 | 17.98 | 122.34 | 68.0 | 24586 | 468 | 1.66 | 53048 | 91.53 | 0.40 | 16.77 | 38.45 |
|  | 12.65 | 0.06 | 0.15 | 13.06 | 12.33 | 110.73 | 17.93 | 85.40 | 69.7 | 27026 | 525 | 1.86 | 63890 | 104.00 | 0.34 | 17.96 | 32.72 |
|  | 14.25 | - | - | - | - | - | - | - | - | - | - | - | - | - | 0.24 | - | 25.39 |
| S7 | 0.40 | 0.31 | 0.28 | 35.96 | 10.13 | 83.17 | 14.83 | 68.75 | 79.68 | 32397 | 539.80 | 2.54 | 51116 | 99.32 | 2.82 | 390.17 | 35.58 |
|  | 0.80 | 0.12 | 0.27 | 40.81 | 5.25 | 72.27 | 14.99 | 61.03 | 62.52 | 24456 | 376.58 | 2.77 | 40168 | 87.19 | 8.94 | 50.01 | 30.80 |
|  | 2.20 |  | 0.13 | 26.77 | 10.53 | 82.16 | 6.95 | 57.66 | 65.86 | 33906 | 408.85 | 2.15 | 59230 | 79.21 | 0.68 | 33.00 | 51.08 |
|  | 3.55 |  | 0.12 | 19.80 | 2.77 | 38.80 | 2.90 | 32.84 | 30.54 | 12235 | 212.80 | 0.97 | 23259 | 47.84 | 21.98 | 19.35 | 42.69 |
|  | 4.90 |  | 0.15 | 22.87 | 9.74 | 73.20 | 7.13 | 55.87 | 63.44 | 28281 | 380.99 | 1.74 | 50830 | 75.55 | 0.71 | 13.45 | 32.83 |
|  | 6.35 |  | 0.17 | 13.38 | 8.70 | 85.56 | 4.76 | 60.05 | 59.34 | 29525 | 392.59 | 1.18 | 48708 | 77.60 | 0.60 | 14.37 | 20.60 |
|  | 7.70 |  | 0.11 | 11.60 | 7.96 | 82.06 | 5.03 | 63.17 | 53.83 | 25167 | 447.14 | 1.00 | 44037 | 71.17 | 0.61 | 21.18 | 20.60 |
|  | 9.10 |  | 0.17 | 13.19 | 6.69 | 76.47 | 5.86 | 67.65 | 68.30 | 26856 | 444.25 | 1.22 | 45482 | 71.19 | 0.75 | 23.56 | 34.20 |
|  | 10.60 |  | 0.15 | 13.87 | 8.25 | 103.12 | 7.54 | 82.20 | 62.09 | 35125 | 503.91 | 1.29 | 57651 | 81.39 | 0.73 | 39.59 | 22.87 |
|  | 12.05 |  | 0.14 | 14.23 | 15.83 | 95.42 | 9.40 | 82.54 | 71.47 | 36671 | 520.42 | 1.61 | 53459 | 94.37 | 0.54 | 15.03 | 23.91 |
|  | 13.15 |  | 0.14 | 13.03 | 7.71 | 90.03 | 8.00 | 77.93 | 67.32 | 34867 | 495.53 | 1.39 | 60172 | 86.30 | 0.61 | 19.78 | 32.50 |
|  | 14.25 |  | 0.14 | 13.91 | 11.17 | 98.66 | 8.19 | 79.49 | 72.72 | 39617 | 558.56 | 1.48 | 67042 | 92.39 | 0.59 | 17.06 | 188.40 |

#### **Supplementary Table S2**. Concentration limits of organic compounds and metals in the soil matrix.

|  | PAHs | PCBs | TPH | As | Cd | Cr | Hg | Ni | Pb | V | Cu | Zn |
| --- | --- | --- | --- | --- | --- | --- | --- | --- | --- | --- | --- | --- |
| Taranto Site threshold (^1^)  [ppm] | 4 | 0.19 | - | 20 | 1 | 160 | 0.8 | 100 | 50 | ‐ | 45 | 110 |
| National Environmental Law threshold (2)  [ppm] | 100 | 5 | 750 | 50 | 15 | 800 | 5.0 | 500 | 1000 | 250 | 600 | 1500 |

#### **References**

1. ICRAM (Istituto Centrale per la Ricerca Scientifica e Tecnologica applicata al Mare. Currently ISPRA). doc. # CII-Pr-PU-TA-valori intervento-01.04 (2004).

2. D.Lgs. 152/2006. Legislative Decree n. 152. Norme in materia ambientale. Gazzetta Ufficiale della Repubblica Italiana n. 88 del 14 aprile 2006 - Supplemento Ordinario n. 96 (2006).
